# A Review on the Progress in Nanoparticle/C Hybrid CMS Membranes for Gas Separation

**DOI:** 10.3390/membranes8040134

**Published:** 2018-12-17

**Authors:** Lin Li, Ruisong Xu, Chengwen Song, Bing Zhang, Qingling Liu, Tonghua Wang

**Affiliations:** 1 Carbon Research Lab., State Key Lab of Fine Chemicals, School of Chemical Engineering, Dalian University of Technology, Dalian 116023, China; lilin121@dlut.edu.cn (L.L.); Ruisong_Xu@126.com (R.X.); chengwensong@dlmu.edu.cn (C.S.); bzhangdut@163.com (B.Z.); liuql@tju.edu.cn (Q.L.); 2 College of Environmental Science and Engineering, Dalian Maritime University, Dalian 116026, China; 3 School of Petrochemical Engineering, Shenyang University of Technology, Liaoyang 111003, China; 4 Tianjin Key Laboratory of Indoor Air Environmental Quality Control, School of Environmental Science and Technology, Tianjin University, Tianjin 300350, China

**Keywords:** Carbon molecular sieve membrane, Gas separation, Hybrid membrane, Polyimide, Nanoparticles

## Abstract

Carbon molecular sieve (CMS) membranes are novel materials derived from the pyrolysis of the polymeric precursors and have a well-developed ultra-microporous structure that can separate small gas pairs with minor difference in diameter, and thus exhibit higher gas permeability and selectivity than polymeric membranes. However, the gas permeability for traditional pure CMS membranes now cannot satisfy the requirements of commercial applications due to their disordered pore structure and high gas molecular diffusion resistance. Incorporating functional materials into membrane precursors to fabricate hybrid CMS membranes has been regarded as an effective way to tune the disordered pore structure of traditional pure CMS membranes, and thus to greatly improve their gas permeability. Many nanoparticles have been tested as the functional foreign materials to fabricate the hybrid CMS membranes with more developed microporous structure and enhanced gas separation performance. This review discusses the hybridized nanoparticle selection and effect of the species, quantities and particle sizes of the foreign materials on CMS membrane characteristics and performance. The function of the materials incorporated inside the hybrid CMS membranes is also analyzed. It is identified that preparation of hybrid CMS membranes provides a simple and convenient route to efficiently improve the trade-off relationship between permeability and selectivity, and to enable the construction of carbon-based composite materials with novel functionalities in membrane science.

## 1. Introduction

Carbon molecular sieve (CMS) membranes, which are derived by the pyrolysis of polymeric precursors under vacuum or inert atmosphere, are novel carbon-based gas separation membranes with a rich ultramicropore structure that can distinguish a gas pair with nearly the same molecule size [1,2,3,4,5,6]. Compared to polymeric membranes for gas separation, CMS membranes exhibit outstanding gas separation performance, such as excellent gas selectivity with high gas permeability, chemical and thermal stability. They can also be used in harsh environments (elevated temperature and high pressure), particularly for their superior separation ability of small molecular gas pairs with similar sizes via the effect of molecular sieving [1,2,3,4,5,6]. Therefore, CMS membranes show great application potential in the gas separation field, including the separation of oxygen and nitrogen from air, carbon dioxide capture from flue gas, propane/propene separation, hydrogen purification and recovery from refinery fuel gas and exhaust gas, methane enrichment and concentration from coal bed gas and biogas, acid gas removal from natural gas, dehydration from fine chemical products and natural gas processing [1,2,3,4,5,6,7]. Moreover, the carbon membrane can also be used in a hydrogen-related membrane reactor to enhance the hydrogen conversion rate due to its much lower cost than a Pd membrane and higher selectivity than ceramic and zeolite membranes [8]. For instance, the hydrogen conversion rate increases from 30% to 70%, because the hydrogen is continually removed during the reaction of dehydrogenation of cyclohexane [9,10]. The methanol conversion rate and hydrogen selectivity in the methanol steam reforming reaction are obviously enhanced in the CMS membrane reactor [11,12].

**Figure 1:**
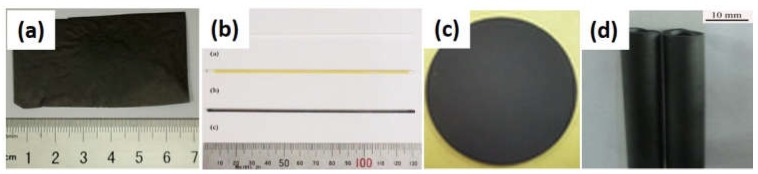
Flat film free-standing carbon molecular sieve (CMS) membrane (**a**), hollow fiber free-standing CMS membrane (**b**), flat supported CMS membrane (**c**) and tubular supported CMS membrane (**d**).

CMS membranes, in general, involve two categories: free-standing (unsupported) and supported CMS membranes (shown in Figure 1). The free-standing CMS membranes (flat film and hollow fiber in Figure 1a,b) have usually been used in lab research o the intrinsic properties of carbon membranes due to their weakness and brittleness under intensity. Supported membranes with a thin top separation layer of CMS membrane, including the flat (Figure 1c) or tube (Figure 1d) membranes, are studied for the purpose of practical application due to their high mechanical strength [13]. The gas separation performance of supported membranes is dependent on the properties of the precursor materials of the CMS membrane as the top layer on the supports.

The structure and property of precursors play an important role in affecting the gas permeability and selectivity of the derived CMS membrane. Although a large number of polymeric materials are good precursors for fabricating polymeric membranes, these are not all suitable for preparing carbon membranes. Only polymeric precursors with high carbon residue and good film-forming property can be used to prepare CMS membranes [1,2,5,6,14]. These polymeric precursors include polyimide (PI) and its derivatives [14,15,16,17,18,19], poly(furfuryl alcohol) [20,21,22], phenolic resins [23,24,25], poly(phthalazinone ether sulfone ketone) [26,27], polyacrylonitrile [28], coal tar pitch [29], cellulose derivatives [30,31], and polymer of intrinsic microporositys (PIMs) [32,33]. In addition, copolymers blended with different polymers, such as poly(benzimidazole) (PBI) blending with PI [34], PPO (polyphenylene oxide) blending with PVP (polyvinylpyrrolidone) [35], and PAN (polyacrylonitrile) blending with PEG (polyethylene glycol) [36], are used to fabricate CMS membranes for improving the structure and properties of the obtained CMS membranes.

Although CMS membranes derived from the above polymeric precursors have exhibited excellent gas selectivity and higher gas permeability than polymeric membranes, their gas permeation can still not satisfy the requirements of commercial applications on a large scale at an economical price [37]. Therefore, enhancing the gas permeation of carbon membranes to meet the requirement of economic feasibility is significant for the commercial application of carbon membranes, and a challenge for the preparation of CMS membranes.

**Figure 2:**
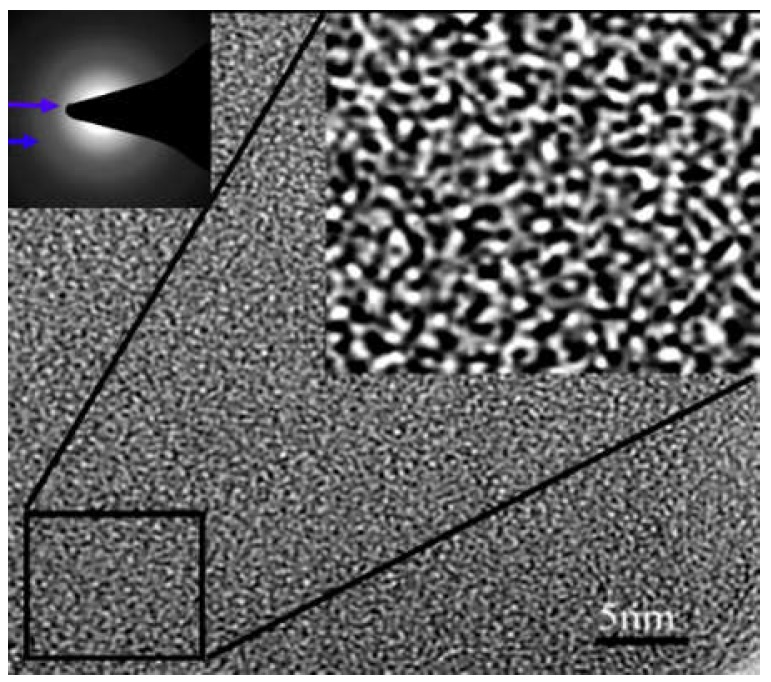
HRTEM image of disordered pore structure in pure CMS membrane [38].

As is known, a carbon membrane has a disordered microstructure. Figure 2 shows the HRTEM (high-resolution transmission electron microscopy) image of PMDA-ODA (poly(pyromellitic dianhydride-4,4-oxydianiline))type PI-based pure CMS membrane [38]. The “white” and “black” stripes coming from different scattering and adsorption strengths of electrons imply the disordered packing of turbostratic carbon sheets and clusters, which form the porous structure of the CMS membrane. Furthermore, the crossing stripes with widths of almost 0.5 nm indicate the gas molecular diffusion routes through the disordered interconnective nano-channels with many twists and turns. The resulting high diffusion resistance of the gas molecules penetrating through the membrane is the key to low gas permeability of the pure CMS membranes [21,38,39]. Hence, reduction of gas diffusion resistance passing through the disordered pore structure of the pure carbon membrane will significantly enhance the gas permeability of carbon membranes.

Incorporating inorganic nanoparticles into the carbon matrix to fabricate hybrid CMS membranes is considered an effective route to tune the disordered pore structure of pure CMS membranes and improve gas permeation via reducing the gas diffusion through the CMS membrane. Research has reported that hybridizing nanoparticles, such as metal clusters [40,41,42,43,44], metal oxides [44,45,46,47], inorganic particles [48,49,50,51], and porous materials [52,53,54,55,56,57,58,59], into the carbon matrix obviously enhances the gas permeability or selectivity of derived carbon membranes.

In the past decade, our group has made efforts to fabricate hybrid CMS membranes by incorporating inorganic nanoparticles, microporous zeolites, mesoporous silica or carbon nanotubes (CNTs) into carbon matrix. The effects of nanoparticles incorporated on the pore structure, properties and gas separation performance of derived hybrid CMS membranes have been systematically investigated. The as-made hybrid CMS membranes have shown a higher gas permeability than pure CMS membranes while maintaining high gas selectivity. In the paper, research on the preparation, properties and gas separation performance of hybrid CMS membranes, of both our group and other researchers (Section 2, Section 3, Section 4 and Section 5), and recent progress on hybrid CMS membranes (Section 6), are reviewed in detail.

## 2. Fe Series Nanoparticles Hybrid CMS Membranes

It was found that preparation of hybrid CMS membranes by doping the metal (Ag, Pt, Pd, etc.), metal salts or the inorganic particles into precursors is effective to improve the gas permeation property of CMS membranes due to the effects of the particles on the different gases and the interfacial gaps between particles and their surrounding carbon matrix. The gas permeability and selectivity of part of the hybrid CMS membranes are listed in Table 1. Barsema et al. incorporated AgNO_3_ or AgAc solutions into polyimide and prepared Ag/C hybrid CMS membranes with higher O_2_/N_2_ selectivity due to the chemical sorption effect of Ag to O_2_ [42,43]. Kim et al. prepared the alkali mental/C hybrid CMS membranes by synthesis of the sulfonated polyimides substituted by Li^+^, Na^+^ or K^+^ [60]. The thermal stability of membranes was enhanced by incorporating the metal ions. The distance between carbon layers was increased with the increase of the diameters of ions, resulting in enhanced gas permeation. Yoda et al. introduced palladium salt into polyimide under supercritical conditions and prepared the Pd/C hybrid CMS membrane with 16 times higher H_2_ permeability than pure CMS membranes due to the effect of Pd to H_2_ [39]. Lie and Hägg added the metal salts, including Cu(NO_3_)_2_·3H_2_O, CaO, MgO, Fe_2_O_3_, AgNO_3_, and Fe(NO_3_)_3_·9H_2_O, into cellulose as precursors and prepared hybrid CMS membranes [45]. The metal/C hybrid CMS membranes showed different gas permeation properties with different metal particles due to the adsorption of different metals to special gases. When the metal particles were added in the form of nitrate, the nitrate could act as a pore-forming agent due to decomposition in carbonization. The related hybrid CMS membranes had higher gas permeability.

Moreover, some gas mixtures could be separated in a magnetic field due to the different forces of the gas with different magnetic properties [61,62]. In this section, a series of Fe-related materials were incorporated into the carbon matrix to prepare the Fe/C hybrid CMS membranes. We hoped the micro-structure of the hybrid CMS membranes could be more developed by utilizing the surface effect and small-size effect to improve the gas permeability of CMS membranes. Meanwhile, the adsorption effect to gas and magnetism of Fe-related particles also helped to improve the gas permeability.

### 2.1. Preparation of the Hybrid CMS Membranes

The hybrid nanoparticles were dispersed into the solvent to produce the suspension under ultrasonic treatment. The PMDA-ODA type polyamic acid (PAA) solution was mixed with the suspension. After high-speed stirring and defoaming, the obtained homogeneous precursor of hybrid CMS membrane was cast on a glass plate and dried to produce the self-standing PAA/nanoparticle hybrid membrane. The thickness of dried polymer membranes was about 100 μm. The hybrid CMS membrane was obtained by carbonizing in the tubular furnace at 600–800 °C for 2 h in flowing argon of 100 mL/min with the heating rate of 2 °C/min. The carbonization temperature of CMS membranes was 700 °C, unless otherwise specified. The thickness of CMS membranes was about 65–75 μm. The content of hybridized nanoparticles equaled the weight of nanoparticles divided by the sum of the weight of pure PAA and the weight of nanoparticles, not including the weight of DMAc (N, N-Dimethylacetamide). 

The gas permeation test method and condition used were the same as Ref. [38]. The other hybrid CMS membranes prepared in this review, except in Section 6, had similar preparation processes.

### 2.2. Ferrocene/PAA Based Hybrid CMS Membrane

**Figure 3:**
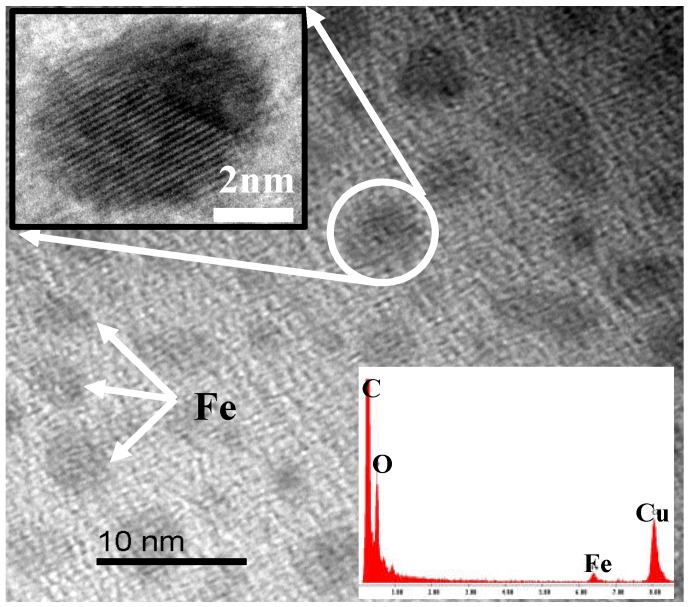
HRTEM image of ferrocene/polyamic acid (PAA)-based hybrid CMS membrane [63].

**Figure 4:**
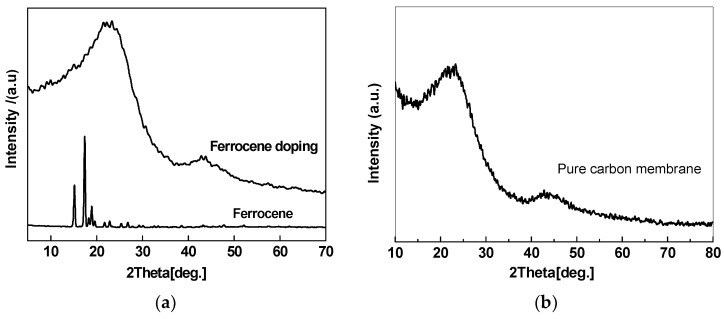
XRD patterns of ferrocene/PAA-based Fe/C hybrid membrane (**a**) and PAA-based pure CMS membrane (**b**) [63].

Ferrocene, a kind of organic transition metal compound with good solubility with DMAc, can form a homogeneous system with PAA solution, which helps improve the dispersion of the α-Fe obtained by the pyrolysis of ferrocene in the CMS membrane. The HRTEM image of the ferrocene/PAA based hybrid CMS membrane is shown as Figure 3. The Fe nanoparticles (identified by the EDX (energy dispersive X-ray) patterns) with diameter of almost 5 nm are dispersed homogeneously in the disordered carbon matrix. The crystal structure of Fe particles with interplanar spacing of 0.206 nm are shown in the insert image. However, the Fe element content in the hybrid CMS membrane is low because of the sublimation of ferrocene. It also could be proved by the XRD (X-ray diffraction) patterns (Figure 4) that the curve of ferrocene/PAA-based Fe/C hybrid membrane is the same as the pure CMS membrane, implying the remaining ferrocene particles, which were coated by the carbon matrix, are very few. The broad (002) diffraction peak at around 24° and (100) diffraction peak at 43° can be observed in these two CMS membranes, implying the carbon matrix presents a turbostratic structure with disordered stacked graphite microcrystals.

The gas separation performance of ferrocene/PAA-based hybrid CMS membrane is shown in Table 2. The ferrocene/PAA-based hybrid CMS membrane presents higher gas permeability due to the more developed pore structure caused by the embedding and pyrolysis of ferrocene than the pure CMS membrane. Gas permeability was improved and selectivity reduced with the increase of content of ferrocene. The best gas separation property in these ferrocene/PAA-based hybrid CMS membranes belonged to the membrane with the content of ferrocene in the precursor of 15 wt%, in which gas permeability is 22 times higher than the pure CMS membrane, and O_2_/N_2_ selectivity is reduced by 40%. When the content of ferrocene reached 20 wt%, the gas selectivity of hybrid CMS membrane fell significantly, even close to 1, because the additional pores became continuous phase in hybrid CMS membranes and weakened the gas molecular sieving effect of carbon matrix.

### 2.3. Fe Series Magnetic Nanoparticle/C Hybrid Membranes

The ferrocene/PAA-based Fe/C hybrid CMS membranes lost most doping particles during the pyrolysis preparation, which weakens the enhancement of gas permeability derived from hybridization because of the sublimation of ferrocene. Three kinds of Fe series magnetic nanoparticle involving Fe_3_O_4_, γ-Fe_2_O_3_ and Zn_0.5_Ni_0.5_Fe_2_O_4_ with similar particle size are adopted as the dopants to prepare the Fe series magnetic nanoparticle/carbon hybrid membranes. We hoped that gas separation performance could be further improved by utilizing the surface effect and small size effect of the nanoparticles and the magnetism functionalization of the hybrid CMS membrane.

**Figure 5:**
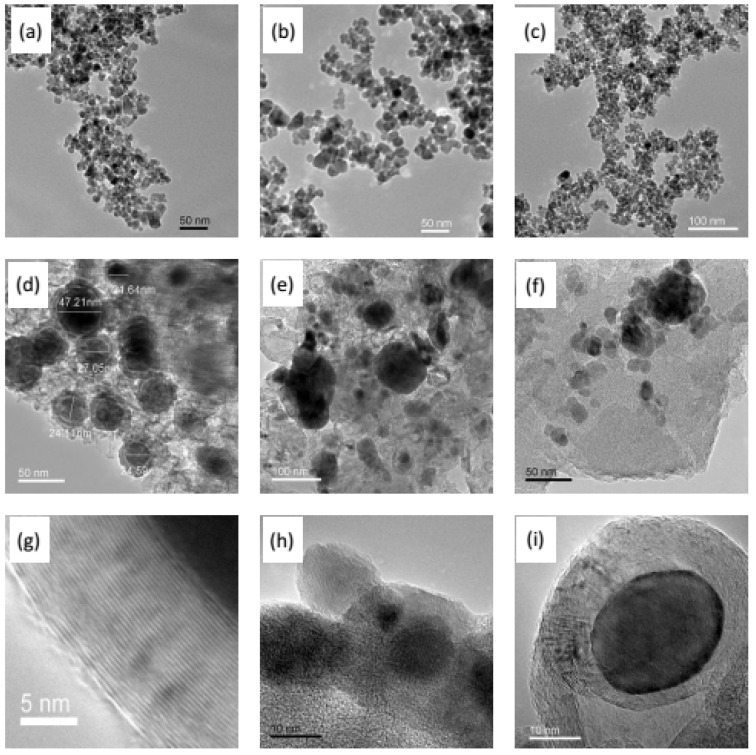
HRTEM images: Fe_3_O_4_ nanoparticles and obtained hybrid CMS membrane (**a**,**d**,**g**), γ-Fe_2_O_3_ nanoparticles and obtained hybrid CMS membrane (**b**,**e**,**h**), Zn_0.5_Ni_0.5_Fe_2_O_4_ nanoparticles and obtained hybrid CMS membrane (**c**,**f**,**i**) (the hybrid CMS membranes with 15% content of Fe series particles and carbonization temperature of 700 °C) [63,64,65].

As shown in Figure 5, the three kinds of Fe series nanoparticles present spherical or irregular spherical shapes. The particle size of Fe_3_O_4_ is almost 10 nm, and is 20 nm for the other two particles. In the Fe_3_O_4_/C hybrid membranes (Figure 5d,g), interfacial pores formed by the micro-phase separation function can be clearly observed between the particles and the carbon matrix. The Fe particles are surrounded by the ordered layered graphite-like carbon, which presents concentric circles with Fe particles due to the catalytic graphitized effect on the carbon matrix from Fe_3_O_4_ particles in the carbonization process. The same catalytic graphitized phenomenon exists in the Zn_0.5_Ni_0.5_Fe_2_O_4_/C hybrid membrane, but not in the γ-Fe_2_O_3_/C hybrid CMS membrane. Additionally, agglomeration happened in the Zn_0.5_Ni_0.5_Fe_2_O_4_/C or γ-Fe_2_O_3_/C hybrid CMS membrane.

**Figure 6:**
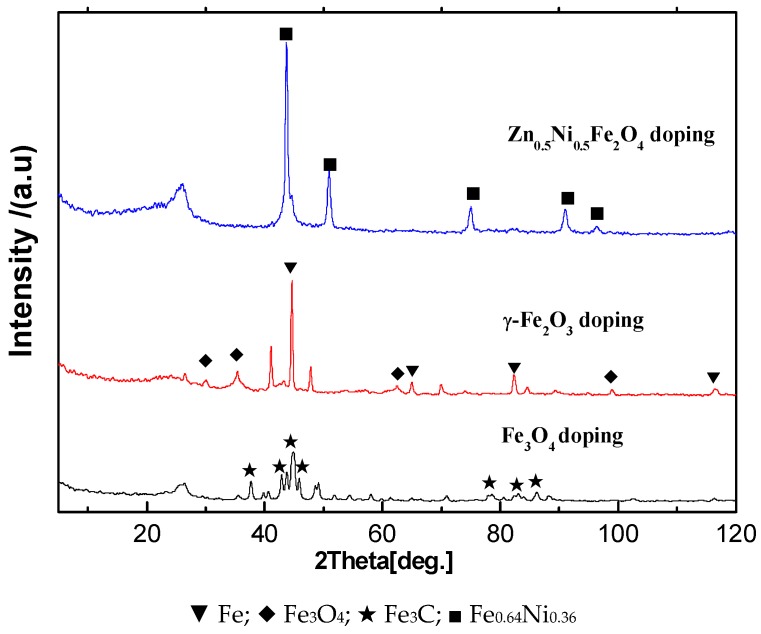
XRD patterns of Fe series magnetic nanoparticle/C hybrid membranes [65].

**Figure 7:**
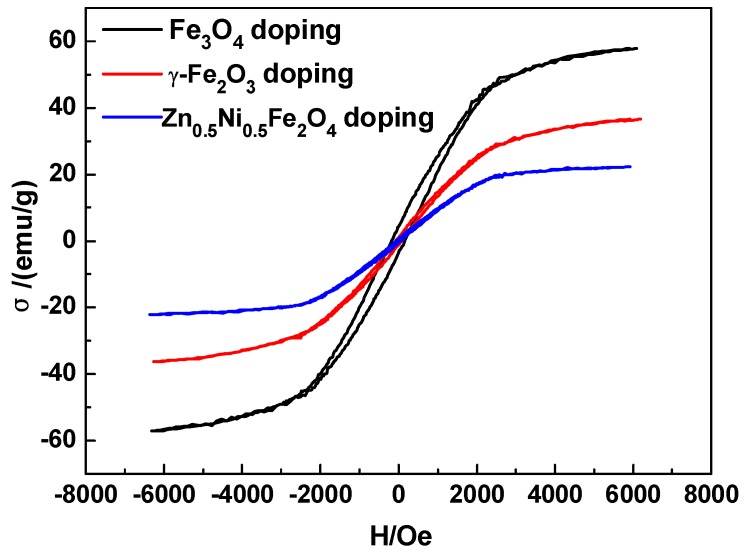
Magnetization curves of Fe series magnetic nanoparticle/C hybrid CMS membranes (content of doping Fe_3_O_4,_ γ-Fe_2_O_3_ or Zn_0.5_Ni_0.5_Fe_2_O_4_ is 15 wt%) [65].

XRD patterns (Figure 6) illustrated the microstructure of Fe series magnetic nanoparticle/C hybrid membranes. The curves of Fe series magnetic nanoparticle/C hybrid CMS membranes present different Fe series crystal characteristic diffraction peaks, as well as the broad (002) diffraction peak of turbostratic carbon structure. Due to the standard spectrum, the main Fe crystalline phases in Zn_0.5_Ni_0.5_Fe_2_O_4_/C and Fe_3_O_4_/C hybrid CMS membranes are Fe_0.64_Ni_0.36_ and Fe_3_C, respectively. The main Fe crystalline phases in γ-Fe_2_O_3_/C hybrid CMS membranes are Fe and Fe_3_O_4_. Three kinds of Fe series magnetic nanoparticle/C hybrid membranes could be attracted by magnet. All the hybrid CMS membranes are soft magnetic materials and presented good magnetic responsibility and low permanence (Figure 7). The hybrid membranes have the highest saturation magnetization of Fe_3_O_4_/C, Fe_2_O_3_/C and Zn_0.5_Ni_0.5_Fe_2_O_4_/C hybrid membranes of 50.24, 36.34 and 22.12 emu/g, respectively. Zn_0.5_Ni_0.5_Fe_2_O_4_/C hybrid membranes exhibit the lowest coercivity and are close to super paramagnetism.

Table 3 shows the gas permeability and selectivity of Fe series magnetic nanoparticle/C hybrid membranes and the metal particle/C hybrid membranes in the reference. The gas permeability of Fe series magnetic nanoparticle/C hybrid membranes is highly improved in comparison with the pure CMS membrane and ferrocene/PAA-based Fe/C hybrid membrane, because the incorporation of the Fe series particles in the carbon matrix developed the pore structure of these hybrid CMS membranes involving the interfacial gaps, observed in Figure 4d–f, constructing the additional gas molecular diffusion routes through the hybrid CMS membranes. Meanwhile, because the doping Fe series magnetic particles do not sublimate as ferrocene in carbonization, more obtained additional pores are maintained. More incorporating particles will form more pores in the hybrid CMS membranes, thus the gas permeability increases and selectivity falls with the increase of the loading of Fe series magnetic nanoparticles in the hybrid CMS membranes.

The Zn_0.5_Ni_0.5_Fe_2_O_4_/C hybrid membrane presents the lowest gas permeability due to the highest agglomeration of Zn_0.5_Ni_0.5_Fe_2_O_4_, which would construct the least number of interfacial pores in the hybrid CMS membranes, and the lowest magnetism of a membrane. The Fe_3_O_4_/C hybrid membrane has the highest H_2_ permeability and similar other gas permeability to the Fe series magnetic nanoparticle/C hybrid membranes with the same Fe series particles content, perhaps because the ordered carbon structure surrounding the Fe particles constructed by the catalytic graphitized effect could highly improve the diffusion of H_2_ molecules with the smallest size, but not to the other gases with bigger molecular size. H_2_ permeability is improved to the greatest degree due to the adsorption from Fe atoms to hydrogen, which is also presented in the ferrocene/PAA-based hybrid CMS membrane. This adsorption effect on gases from metals has been commonly adopted in enhancing the gas permeation or separation of the metal/C hybrid CMS membranes [40,41,42,43,45,60]. The Ag/C hybrid CMS membranes [42,43] showed a higher oxygen permeation (Table 3), because the adsorption–diffusion process happened easily in the membranes. Oxygen is easy to be adsorbed on the active site of Ag and desorbed in the interfacial pores existing around Ag clusters with the diameter of 50 nm. However, the opposite phenomenon in the Ni or Pt/C hybrid CMS membranes, chemical adsorption of metals on the H_2_, happened in the penetration, but desorption was difficult due to lack of interfacial pores. Therefore, Ni or Pt/C hybrid CMS membranes presented much lower H_2_ permeability and extremely higher H_2_/N_2_ selectivity [40,41]. Hence, gas permeation and separation could be highly improved by effectively controlling the adsorption effect of metal particles and pore structure of the hybrid CMS membranes.

Compared to the pure CMS membrane, Fe series hybrid CMS membranes showed significantly increased gas permeability, especially H_2_ permeability, due to the adsorption effect of iron to H_2_. Sublimation of ferrocene particles led to a relatively low permeability of hybrid CMS membranes. The phase structure and morphology of Fe_3_O_4_, γ-Fe_2_O_3_ and Zn_0.5_Ni_0.5_Fe_2_O_4_ changed in pyrolysis and their particle sizes increased. The catalytic graphitization appeared in the Fe_3_O_4_ or Zn_0.5_Ni_0.5_Fe_2_O_4_ related hybrid CMS membranes with much higher H_2_ permeability, in which H_2_ more easily penetrates through the space between graphite-like layers around the particles. The Fe/C hybrid CMS membrane has good application prospects in the field of hydrogen separation and enrichment.

Moreover, the hybrid CMS membranes with Fe_3_O_4_, γ-Fe_2_O_3_ or Zn_0.5_Ni_0.5_Fe_2_O_4_ presented both good magnetic response and high gas permeability. However, the influence of the magnetic property on the gas permeability is overshadowed by the larger influence from the adsorption effect and interfacial pores of metal particles. Thus, this work only made a preliminary exploration of magnetic CMS membranes. The related research work still needs to be further studied.

## 3. Microporous Zeolite/C Hybrid Membranes

The nanoparticles/C hybrid CMS membranes, including Fe and the other nanoparticles such as metal (Ag, Pt, Pd, etc.), metal salts or the inorganic particles, in Section 2, have exhibited the obvious enhancement of gas permeability, which mainly depends on the increase of interfacial pores and adsorption effect of metal particles. The particle sizes and properties of nanoparticles greatly affect the gas separation performances of derived hybrid CMS membranes because they are solid without internal pores. We assumed that it should be possible to further enhance the gas permeability of the hybrid CMS membranes if the nanoparticles possess a well-distributed inner pore structure, which will act as fast channels for gas diffusion. In this section, nanoparticles with the inner pore structure were incorporated into the carbon matrix to investigate the effect of inner pore structure on the gas permeability of hybrid CMS membranes.

**Figure 8:**
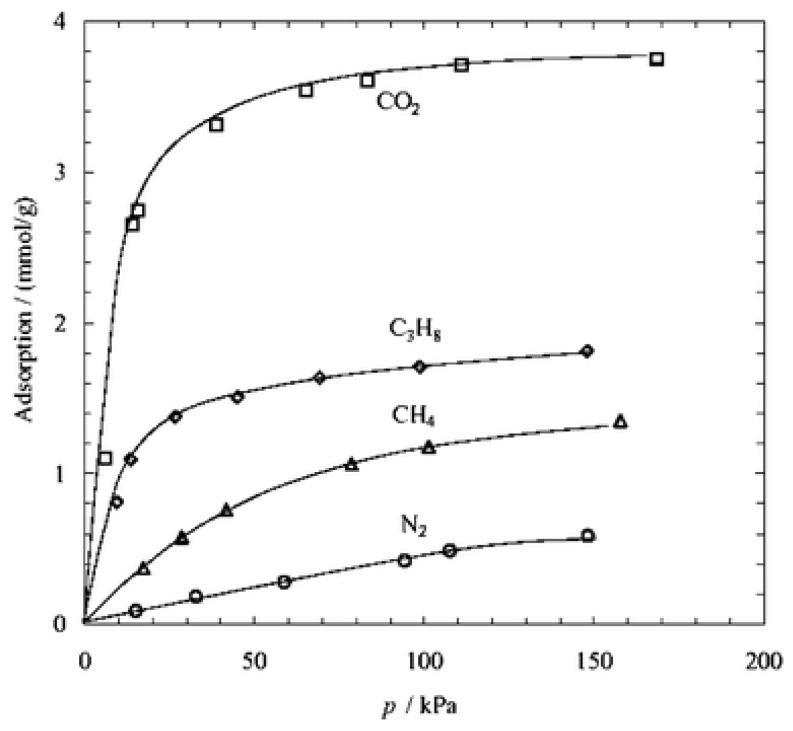
Adsorption isotherms of CO_2_, N_2_, CH_4_ and C_3_H_8_ measured at 25 °C on zeolite T powder [72].

Zeolite is a kind of cage-type or pore-type microporous crystal material composed of silica-oxygen tetrahedron and alumina-oxygen tetrahedron interlinked by oxygen bridge. Zeolites with ordered inner channels can adsorb and separate gas or liquid molecules due to the channel size, which is widely used in the fields of adsorption, separation and catalysis [66,67,68,69,70,71,72,73]. For example, zeolite T membrane can enrich CO_2_ by separating the mixtures of CO_2_/CH_4_ or CO_2_/N_2_ because of the strong adsorption effect on the CO_2_ from zeolite T (Figure 8) [72].

Compared to the disordered ultramicropore structure in CMS membranes, the ordered inner channels in the zeolite particles will reduce the gas diffusion resistance. In this section, a series of zeolite particles were incorporated into polymeric precursor to fabricate zeolite/C hybrid CMS membranes, with the aim of applying the high permeability attributes of zeolites and the high selectivity attribute of the carbon matrix to the zeolite/C CMS membranes. The influence of zeolite type, content, particle size and carbonization conditions on gas separation performance of zeolite/C hybrid CMS membranes was systematically investigated.

**Figure 9:**
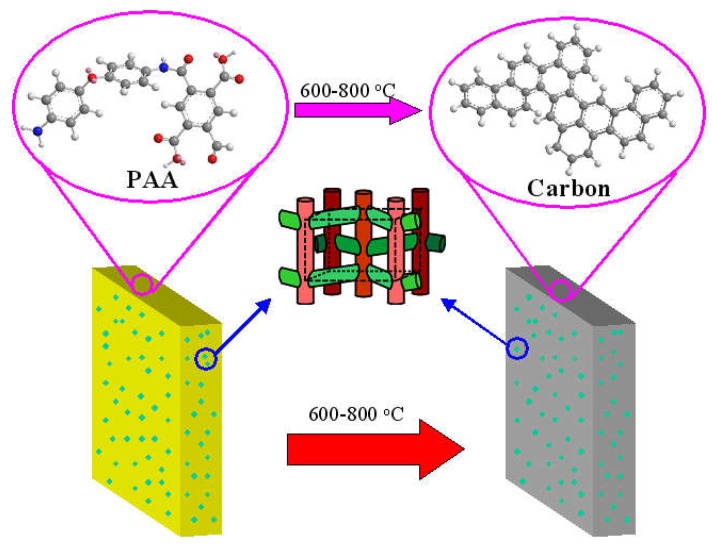
The structure model of zeolite/C hybrid membrane (e.g., ZSM-5/C hybrid membrane) [56].

The structure model of a typical zeolite/C hybrid membrane (ZSM-5/C hybrid membrane as an example) is shown as Figure 9. The gas permeation property of hybrid CMS membranes would be enhanced by utilizing the rapid adsorption and diffusion effect on the small molecular gases from the inner channels of zeolites as well as the high gas molecular sieving effect of carbon matrix [56].

### 3.1. The Effect of Different Zeolites on the Structure of the Hybrid CMS Membranes

**Figure 10:**
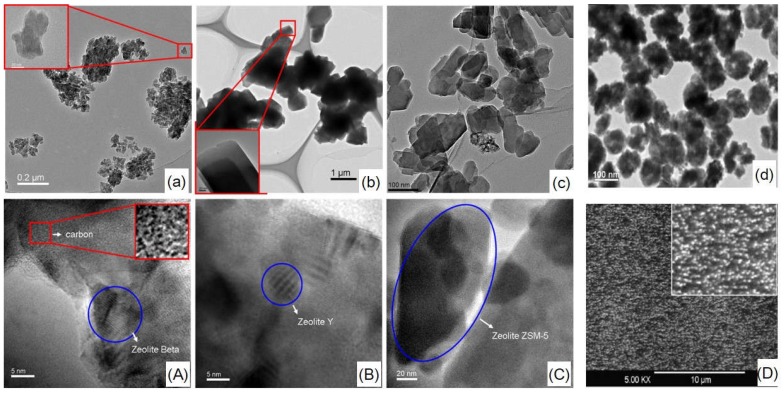
HRTEM and SEM images of zeolites: β (**a**), Y (**b**), ZSM-5 (**c**) [59] and zeolite L [54] (**d**) and related hybrid CMS membranes: β/C (**A**), Y/C (**B**), ZSM-5/C (**C**) [59] and zeolite L/C [54] (**D**).

**Figure 11:**
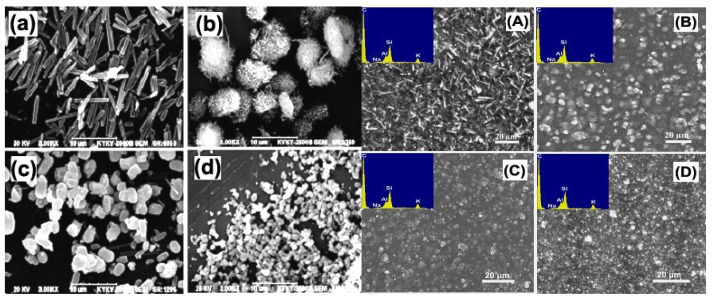
SEM images of zeolite T: (**a**) T-8, (**b**) T-6, (**c**) T-3, (**d**) T-0.5 and T/C hybrid membranes: (**A**) TC-8, (**B**) TC-6, (**C**) TC-3, (**D**) TC-0.5 [57].

The micro-morphology of zeolites and related hybrid CMS membranes is illustrated by the HRTEM and SEM images (Figure 9 and Figure 10). Zeolite β, Y and ZSM-5 with different crystal sizes present similar agglomeration size of about 200 nm (Figure 10a–c). The particle size of zeolite L [54] is as small as 100 nm. The zeolite T with different shape and particle sizes—T-8: 8 µm, T-6: 6 µm, T-3: 3 µm, and T-0.5: 0.5 µm—have homogeneous particle sizes (Figure 11a–d). In the HRTEM images and SEM images of hybrid CMS membranes, all the zeolites dispersed well in the hybrid CMS membranes without large agglomeration and cracking (Figure 10A–D); only the zeolite T-0.5 with the smallest particle size existed as small agglomerations (Figure 11d). The zeolites embedded in the carbon matrix could maintain their shape intact and uniform inner channels (Figure 10A–C and Figure 11A–D). The carbon matrix presents the turbostratic carbon structure (disorderly staggered black and white stripes in the red box of Figure 10A, the same as in Figure 1). The interfacial gaps exhibited as the white lines between the zeolite particles and the carbon matrix, which was rooted in the micro-phase separation effect of the zeolites on carbon matrix, can be clearly observed from Figure 10A–C, but not from Figure 10D.

The structure and property of related zeolite/C hybrid membranes would be greatly impacted by the membrane preparation conditions, such as the content of zeolite and the carbonization process, and the parameters of zeolites, including particle size, porous structure, dispersion and thermal resistance.

### 3.2. The Effect of Zeolite Content on the Gas Separation Performance of Hybrid CMS Membranes

Zeolite β and Y were chosen to prepare hybrid CMS membranes with zeolite content of 5–25% in the precursor for exploring the effect of zeolite content on the hybrid CMS membranes. Hybrid CMS membranes present much higher gas permeability than pure CMS membrane (Table 4). Gas permeability is further improved with the increased content of zeolites due to more ordered inner channels and more interfacial pores formed from more zeolite loading amounts, while the selectivity is reduced, except for the case of O_2_/N_2_ in which is first increased and then decreased, which suggests that larger interfacial gaps should be formed. This indicates that more zeolite particles in the carbon matrix might be conductive to the permeation of the larger gas molecules.

Most of the literature on preparation of hybrid CMS membranes is aimed at improving selectivity and sacrificing partial permeability. Therefore, the preparation of mixed matrix membranes (precursor membranes) and hybrid CMS membranes in the literature has always tried to avoid the formation of interfacial pores to reduce selectivity. For example, the metal particle (Pt, Pd or Ni)/C hybrid CMS membranes [40,41] and the zeolite Y/C hybrid CMS membrane [52], presented a reduced CO_2_ permeability by more than 50% and selectivity increased by more than two times compared to the pure CMS membrane (Table 4).

However, we believe that it is possible to improve the permeability of CMS membranes while maintaining their selectivity, which would effectively promote the practical application of CMS membranes. This is also the main purpose of this review and our research. Therefore, hybrid carbon membranes in our group were maintained with the interfacial pores that would promote gas penetration to improve permeability. The size of the interfacial pores can be changed due to the size of the doped particles and the surface chemical structure of the particles. Thus, the permeability of the hybrid CMS membranes can be adjusted accordingly.

### 3.3. The Effect of Zeolite Particle Size on the Structure and Property of Hybrid CMS Membranes

The existence of zeolites in hybrid CMS membranes involves single crystal dispersion and agglomeration dispersion. Gas separation performance of the zeolite/carbon hybrid membranes could be tuned via the change of agglomerated cluster size and single crystal diameter of doped zeolite particles.

The micromorphology of adopted single crystal zeolite T and relevant hybrid CMS membranes are illustrated in Figure 10. The gas separation performance of hybrid CMS membranes with different size single crystal is shown in Table 5. The gas permeability in the CMS membranes is enhanced and selectivity is reduced with the increase of single crystal diameter of zeolites. This implies the formation of a larger and more developed pore structure, especially of interfacial pores, appearing in the hybrid CMS membranes by doping bigger single crystal zeolites. The shape of zeolite T also has a weaker effect on the gas separation performance of hybrid CMS membrane than the effect of the particle size.

**Figure 12:**
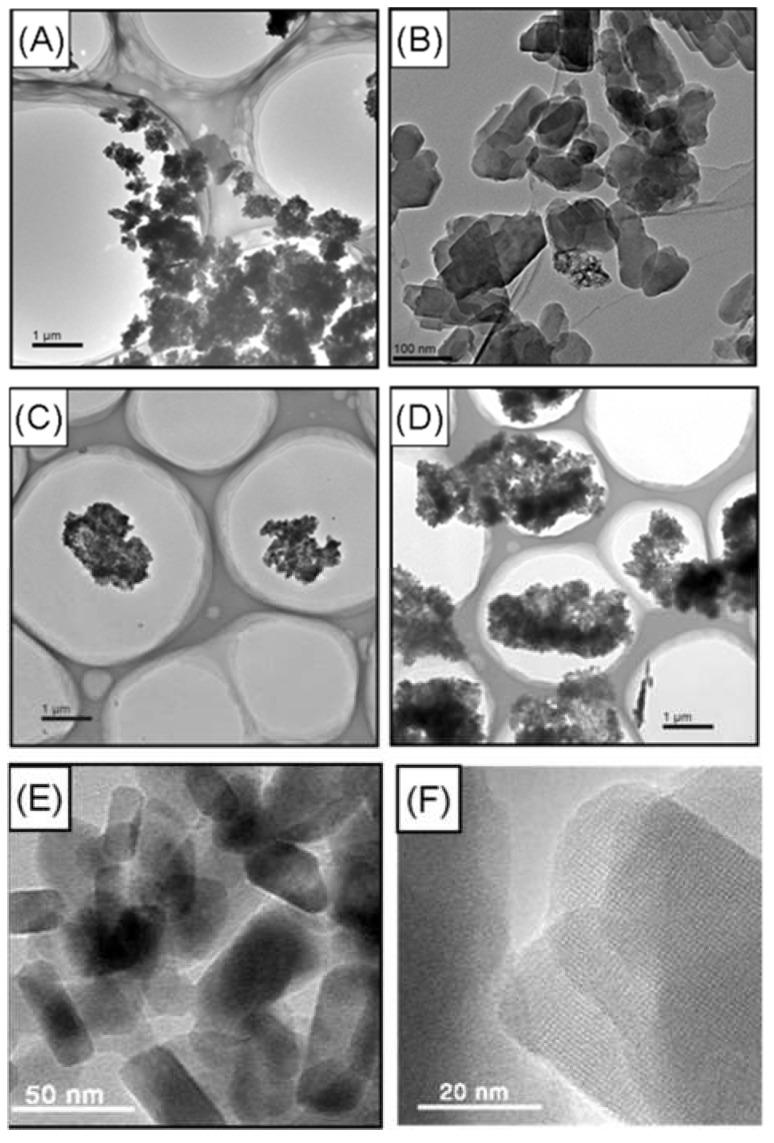
The HRTEM images of agglomerated zeolite ZSM-5 with different particle size: (**A**) 0.2–4 μm [59], (**B**) 0.2 μm [59], (**C**) 1 μm [59], (**D**) 4 μm [59], (**E**,**F**) 20–50 nm [55].

The HRTEM images of zeolite ZSM-5 with different agglomeration size are shown in Figure 12. The gas separation performance of the prepared hybrid CMS membranes is illustrated in Table 6. The gas permeabilities of H_2_, CO_2_ and O_2_ decrease dramatically with the size of the zeolite clusters increasing from 20–50 nm to 0.2 μm and slightly change from 0.2 μm to 4 μm, but the N_2_ permeability increases clearly with the size of the zeolite clusters increasing from 0.2 μm to 4μm which causes a reduction in N_2_-related selectivity. The molecular sieving effect in the hybrid CMS membranes with larger zeolite agglomeration was weakened more significantly. Nevertheless, doping the zeolite with small agglomerating clusters (20–50 nm) into the precursor should create much more interfaced pores (the interfaced gaps or gas permeation channels) between the zeolite and carbon matrix in the hybrid CMS membrane, which exhibited much several times higher gas permeability than the other hybrid CMS membranes, as well as similar gas selectivity.

**Figure 13:**
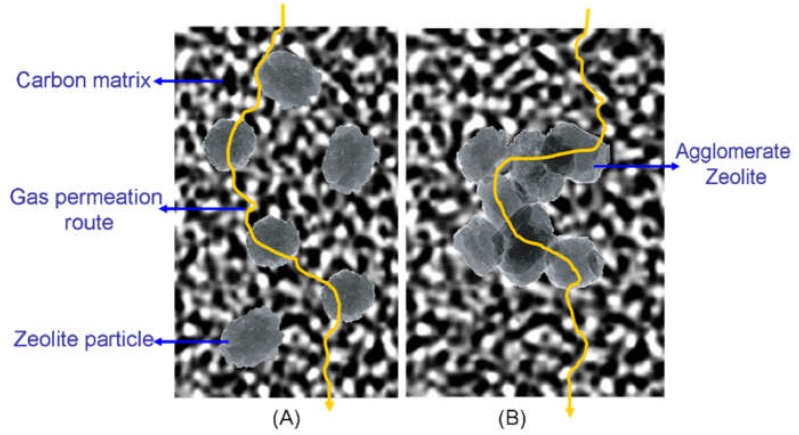
The model of the gas pathway through the zeolite/C hybrid membranes [59]. (**A**) well dispersed zeolite particles in the carbon matrix; (**B**) agglomerate zeolites in the carbon matrix.

In the model of the gas penetrating pathway through the zeolite/C hybrid membranes (Figure 13), the single crystals are dispersed well in the carbon matrix (Figure 13A), and the orifices of zeolites are easily modified by the carbon matrix. Thus, the derived CMS membrane shows much greater gas permeability while maintaining selectivity. However, the orifices of zeolites in the center of the bigger zeolite cluster agglomerated could hardly be modified (Figure 13B), where the molecular sieving effect of carbon matrix is weaker. Moreover, the interfacial pores are bigger in hybrid CMS membranes with bigger cluster agglomerated zeolite, also helping to improve the gas permeability of all the permeating gases and to reduce selectivity.

### 3.4. The Effect of Carbonization Temperature and Zeolite Channel Integrity on the Structure and Property of the Zeolite/C Hybrid Membranes

Carbonization temperature is one of the most important preparation parameters of CMS membranes, and impacts greatly on the structure and property of CMS membrane. Zeolite Y and β with different thermal resistances were chosen to prepare the hybrid CMS membranes and analyze the effect of carbonization temperature on the zeolites and hybrid CMS membranes.

**Figure 14:**
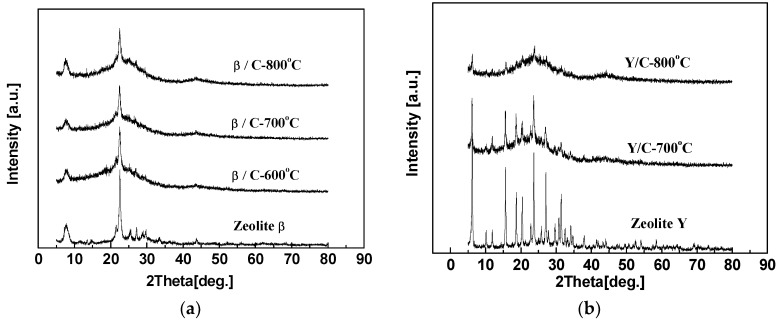
XRD patterns of zeolite β/C (**a**) and Y/C (**b**) hybrid membranes prepared at different pyrolysis temperatures [59].

The XRD patterns of hybrid CMS membranes carbonized at 600, 700 or 800 °C are shown in Figure 14. The curves of hybrid CMS membranes all present characteristic diffraction peaks of carbon matrix and the strong characteristic peaks of doping zeolites, except the Y/C hybrid membrane carbonized at 800 °C. It implies that the crystal structure and ordered inner channels of zeolite Y was destroyed due to the low thermal resistance caused by low Si/Al. With the carbonization temperature increasing, the (002) peak in the hybrid CMS membranes shifted to a high degree and (100) peak intensity became larger, implying the carbon matrix became denser.

The pure CMS membranes and hybrid CMS membranes present an increasing gas selectivity and a reducing permeability by improving the carbonization temperature. In particular, the permeability of pure CMS membranes achieved a maximum at 700 °C and then reduced (Table 7). During the pyrolysis process, the carbon structure in the membrane gradually formed and constructed the micropore structure in the carbon matrix to promote gas permeation. When the temperature reached 800 °C, thermal polymerization was more dominant than thermal decomposition in the pyrolysis process of the carbon matrix. The denser carbon structure with higher diffusion resistance (Figure 14) reduced gas permeation and increased selectivity both in the pure CMS membranes and the hybrid CMS membranes. Moreover, the interfacial gaps between doping particles and the carbon matrix also shrank due to the increasing carbonization temperature, impeding gas molecules in penetrating across the membrane, especially for big gas molecules such as N_2_, and increasing the gas selectivity. Comparing the two kinds of zeolite hybrid CMS membranes, the membranes with the same carbonization temperature presented similar gas separation performance, except the YC-800 presented a lower permeability due to the low thermal stability of zeolite Y. The inner ordered structure was destroyed (Figure 14b).

**Figure 15:**
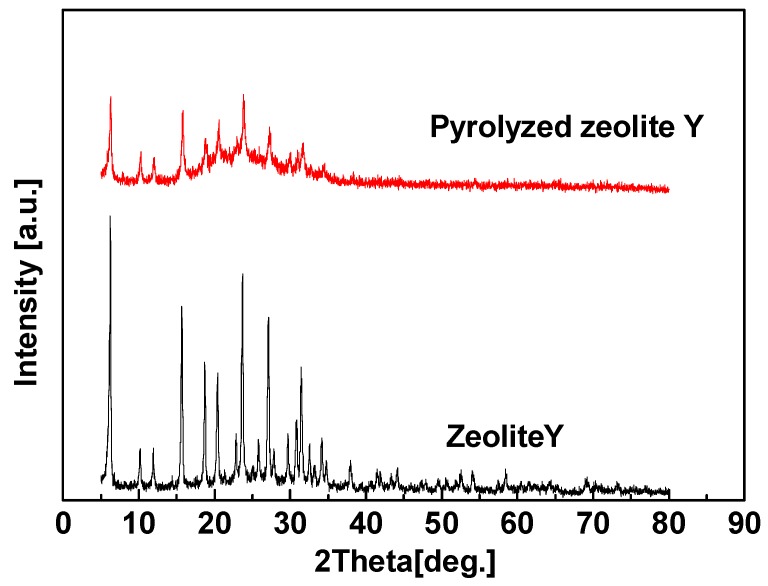
XRD patterns of Zeolite Y and Zeolite Y pyrolyzed at 800 °C [59].

Zeolite Y (heated at 800 °C for 6 h) with destroyed crystal structure (Figure 15) was used to prepare hybrid CMS membrane for proving the enhancement of gas penetration from zeolite channel integrity. Compared with the hybrid CMS membrane with intact zeolites, the gas permeability of zeolite Y (pyrolyzed)/carbon hybrid membrane is obviously reduced, but still higher than pure CMS membrane (Table 8). Although the inner channels of zeolites were destroyed, the interfacial pores still could be formed and accelerated gas permeation. Therefore, the intact ordered inner pore structure of zeolites and interfacial pores are the key factors to enhance the gas separation performance of the zeolite/carbon hybrid membranes.

Similar to Fe/C hybrid CMS membranes, zeolites/C hybrid CMS membranes show much higher gas permeability than the pure CMS membrane due to the inner channels of zeolites and interfacial pores that are formed. Zeolite T/C hybrid CMS membranes present higher CO_2_ permeability owing to the adsorption effect. The structure and permeation performance of hybrid CMS membranes can be tuned by optimizing the parameters, such as loading of zeolites, particle size, carbonization conditions and loading content.

## 4. Ordered Mesoporous Silica/C Hybrid CMS Membranes and CNTs/C Hybrid CMS Membranes

The high gas permeability of zeolites /C hybrid CMS membranes indicates that the incorporation of the zeolites with the microporous structure into the carbon matrix will greatly enhance the gas permeation and maintain a relatively high gas selectivity, implying a significant effect of the inner channels of doping particles on gas permeability. In this section, the effects of internal pore size of doping nanoparticles on the gas permeability of the carbon membrane are investigated and discussed by hybridizing the nanoparticles with a mesoporous structure, such as ordered mesoporous silica or carbon nanotubes (CNTs), into the carbon matrix to prepare the mesoporous particle/C hybrid CMS membranes.

### 4.1. Ordered Mesoporous Silica/C Hybrid CMS Membranes

From the first aluminosilicate ordered mesoporous material, M41S [74,75], to the those that subsequently appeared, such as SBA-n, MSU series, HMS, FSM-16, MAS series, and JLU series, mesoporous materials have attracted great attention in the membrane separation, catalyst, molecular engineer, etc., fields [76,77,78,79,80,81,82,83]. These novel mesoporous materials could not only break the limits of porous range in zeolites, but also exhibit outstanding properties different from those of macroporous materials due to the quantum confinement effect, macroscopic quantum effect and dielectric confinement effect of mesoporous materials.

**Figure 16:**
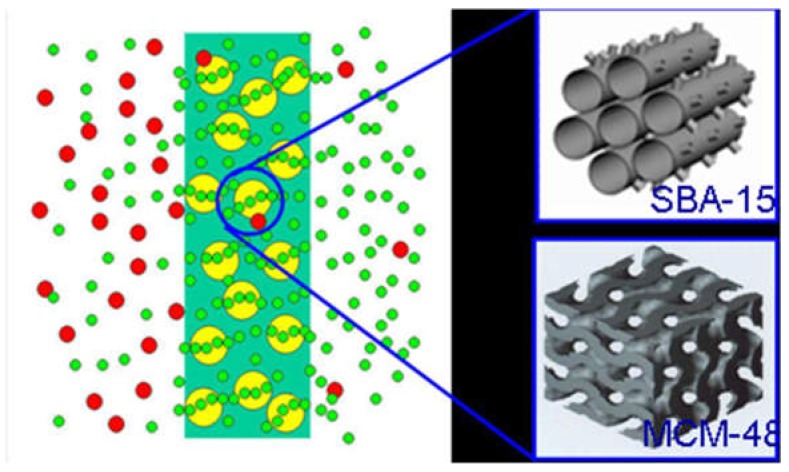
The structure model of ordered mesoporous silica/C hybrid CMS membrane [38].

More importantly, the diffusion of small molecular gases, such as H_2_, CO_2_, O_2_, N_2_ and CH_4_, through the inner channels of mesoporous materials is several orders of magnitude higher than in the microporous zeolites [84]. In this part, the ordered mesoporous material/C hybrid membrane materials were designed (Figure 16) and fabricated by incorporating the ordered mesoporous silica SBA-15 or MCM-48 into the carbon matrix.

**Figure 17:**
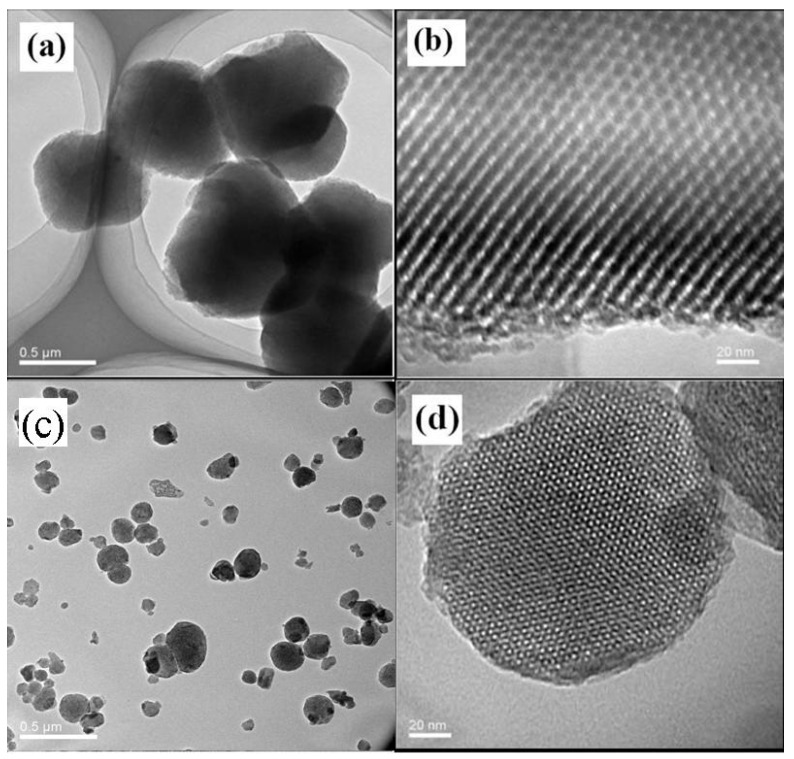
TEM (transmission electron microscopy) images of the as-made SBA-15 (**a**,**b**) and MCM-48 (**c**,**d**) [38].

**Figure 18:**
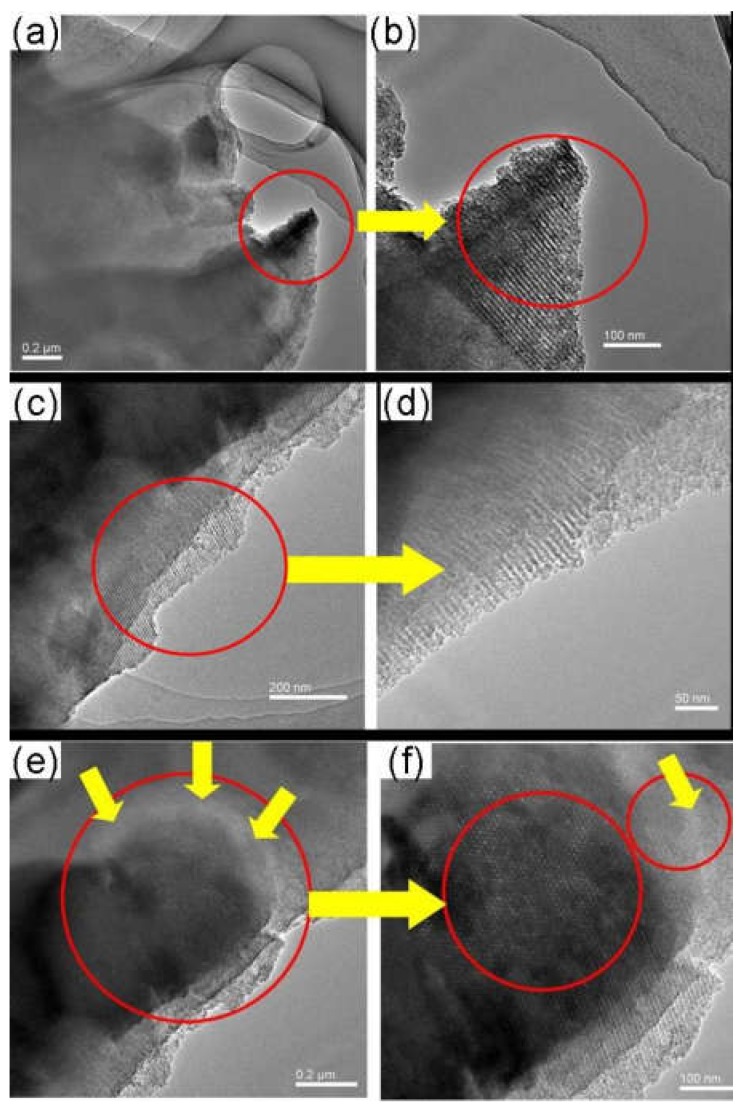
TEM images of the as-made SBA-15/C functional membranes [38].

**Figure 19:**
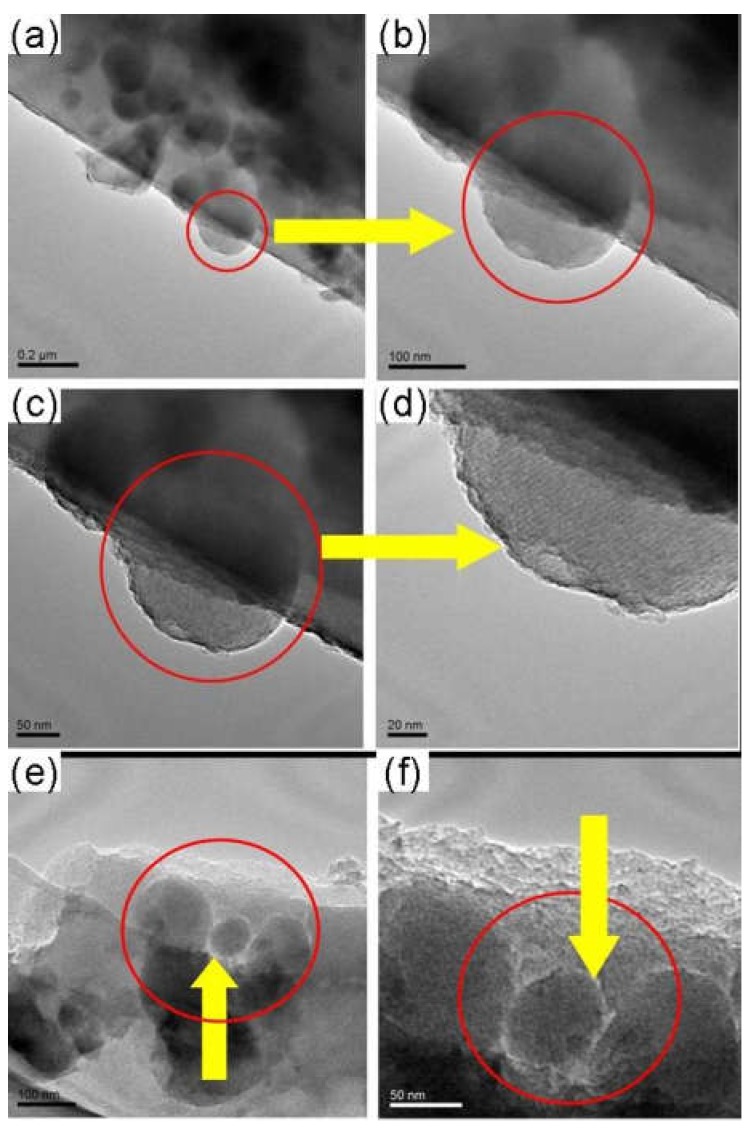
TEM images of the as-made MCM-48/C functional membranes [38].

The ordered microstructure of SBA-15 with the particle size of 500 nm and a two-dimensional ordered structure, and MSM-48 with the particle size of 100–200 nm and three-dimensional cubic structure particles can be observed in Figure 17. Moreover, the nanoparticles with intact ordered structures disperse in the carbon matrix homogenously, and the interfacial pores (lighter lines) exist between the nanoparticles and around the carbon matrix in Figure 18 and Figure 19.

**Figure 20:**
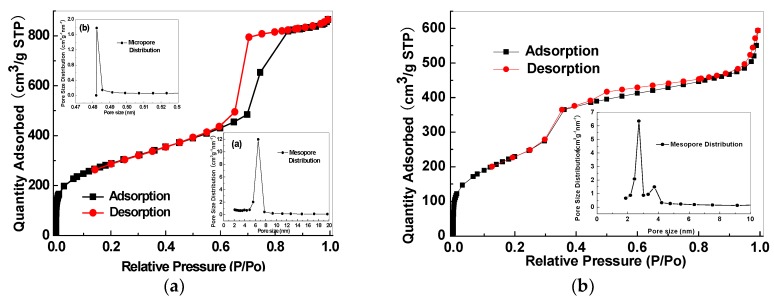
Nitrogen adsorption isotherms and pore size distributions of (**a**) SBA-15 and (**b**) MCM-48.

**Figure 21:**
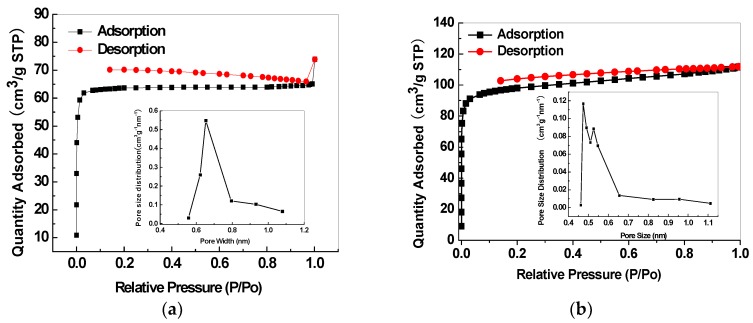
Nitrogen adsorption isotherms and pore size distributions of the SBA-15/C (**a**) and MCM-48/C (**b**) hybrid membranes [38].

The porous structure properties of the mesoporous silica and relevant CMS membranes were analysed by N_2_ adsorption isotherm and pore size distribution. The adopted SBA-15 presents both micro- and mesoporous structures with the average micropore size of 0.485 nm and an average mesopore size of 5.8 nm (Figure 20a). MCM-48 has a mesoporous structure with an average size of 2.7 nm (Figure 20b). The SBA-15/C (Figure 21a) and MCM-48/C (Figure 21b) hybrid membranes have only microporous structures and micropore volumes of 0.1513 cm^3^/g and 0.1807 cm^3^/g, respectively, calculated by Dubinin-Astakhov equation, and mean pore sizes of 0.65 nm and 0.52 nm, respectively, calculated by the H-K method.

The MCM-48/C hybrid membrane presented more than two times higher gas permeability and slightly improved selectivity compared to the SBA-15/C hybrid membrane (Table 9), because more developed micropores and smaller pore size in the MCM-48/C hybrid membrane implies higher gas permeating capability. Additionally, MCM-48 with smaller particle size than SBA-15 could produce more gas penetrating channels under the same doping content in the related hybrid CMS membranes, which could further accelerate gas penetration in the MCM-48/C hybrid membrane.

### 4.2. CNTs/C Hybrid CMS Membranes

CNTs are potential hybridized particles with which to fabricate mixed matrix membranes [85] and hybrid CMS membranes with high gas separation performance [86,87,88], attributed to the rapid transport of gases through CNTs with low transport resistance. However, the systematic study of CNTs/C hybrid CMS membranes is lacking. In this section, a series of CNTs/C hybrid CMS membranes were prepared by carbonizing the precursors involving PMDA-ODA type PAA and CNTs [89]. Also, the effects of CNT properties, such as CNT type, concentration, length and diameter, on the property of hybrid CMS membranes were investigated [89].

**Figure 22:**
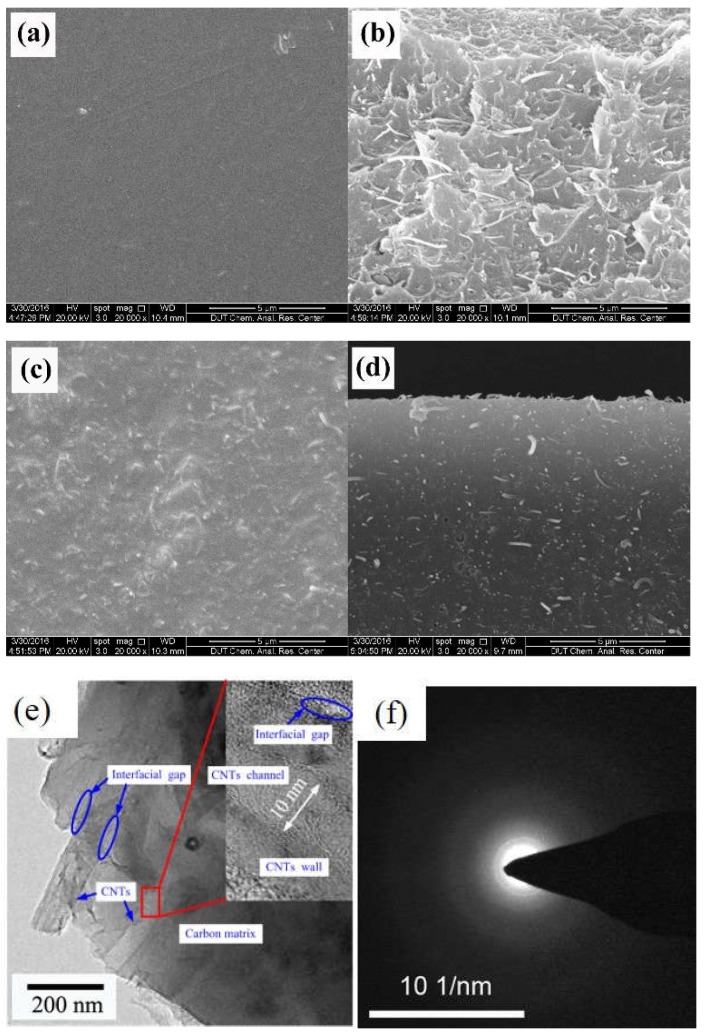
Micromorphology of the obtained CNT-related hybrid membranes. (**a**) Surface and (**b**) cross-section SEM images of CNT/PAA composite membranes; (**c**) surface and (**d**) cross-section SEM images of CNT/C hybrid membranes; (**e**) HRTEM image of CNT/C hybrid membrane; and (**f**) selected area diffraction (SAED) pattern of MWCNTs [89].

The SEM and HRTEM images of the obtained CNT-related hybrid membranes are shown in Figure 22. The CNT particles dispersed homogeneously in the form of the single tube in both the matrixes of CNT/PAA membrane (Figure 22b) and the related hybrid CMS membranes (Figure 22d,e) without cracks or defects in the surface of membranes (Figure 22a,c). The interfacial gaps between the CNT and carbon matrix can be observed in Figure 22e. Additionally, the MWCNTs with low graphite degree could be implied.

The effects of CNTs with different properties on gas permeation performance are shown in Table 10. Gas permeability in hybrid CMS membranes is much higher than for pure CMS membranes, especially CO_2_ permeability, which is even higher than H_2_ permeability. CO_2_/N_2_ selectivity is also higher due to the unique adsorption and capture effects of CNTs to carbon dioxide molecules. The interfacial pore size is the principle affecting factor on the gas permeation property of the CNT/C hybrid CMS membranes. The gas permeability presented by MWCNT/C hybrid CMS membrane is higher than SWCNT/C hybrid CMS membranes due to the larger interfacial pores between the MWCNTs with a bigger size and carbon matrix. For the same reason, MWCNTs with greater length and diameter were conducive to produce hybrid CMS membranes presenting higher gas permeability. Simultaneously, higher permeability in hybrid CMS membranes had been accompanied by lower gas selectivity because of the larger interfacial pores which would weaken the molecular sieving effect of the hybrid CMS membranes. However, the difference is that longer MWCNTs with the same diameter related hybrid CMS membranes present higher CO_2_/N_2_ selectivity due to more CO_2_ adsorption sites in longer CNTs, which accelerate CO_2_ permeation. After acid treatment, some MWCNTs would be opened up to afford more gas permeation routes and loaded with polar carboxylic acid groups and hydroxyl groups, which enhance CNT compatibility with the precursor matrix and lead to a more compact hybrid CMS membrane. As a result, the MWCNT (acid treated)/C hybrid CMS membranes showed higher gas permeability and selectivity.

### 4.3. Comparison and Analysis of the Gas Separation Property of Hybrid CMS Membranes

The gas permeability and selectivity of the representative hybrid CMS membranes are also listed in Table 11. The gas permeabilities in all the hybrid CMS membranes are highly improved. Compared to the zeolite/C hybrid membranes, the ordered mesoporous silica/C hybrid membranes, especially the MCM-48/C hybrid CMS membrane, present lower H_2_ permeability and higher permeabilities of the other gases because the width of ordered channels in mesoporous silica is 3–5 nm and larger than the 0.5–0.8 nm of microporous zeolite, predicating higher acceleration in permeation of the bigger molecular gases. The Fe_3_O_4_/C and ZSM-5/C hybrid CMS membranes with the smallest particle sizes of 20–50 nm have the highest gas permeability due to the biggest number of additional channels. The Fe_3_O_4_/C hybrid membrane presents higher H_2_ permeability than the ZSM-5/C hybrid CMS membrane due to the unique adsorption from Fe atom to hydrogen molecules. However, other gas permeability in the Fe_3_O_4_/C hybrid membrane is lower than in the ZSM-5/C hybrid CMS membrane with lower particle content due to ZSM-5 owing inner channels. Moreover, CNT/C hybrid CMS membranes present the highest CO_2_ permeability, even higher than H_2_, due to the strong affinity from CNTs to CO_2_ molecules. Among all characteristic properties of doping nanoparticles, the minimum particle size, the most developed inner channels and unique adsorption effects on some gas molecules are the most effective parameters for the acceleration of gas permeation of hybrid CMS membrane.

## 5. The Function of Inorganic Nanoparticles to Enhance the Gas Permeability of Hybrid CMS Membrane

**Figure 23:**
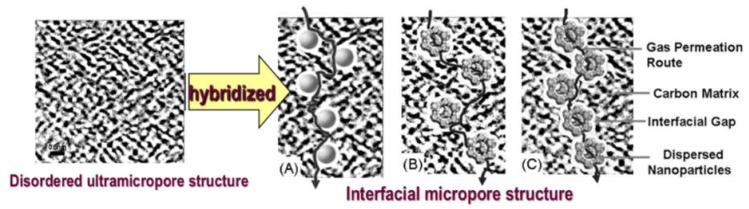
The structure and gas diffusion routes model of inorganic nanoparticles/C hybrid membranes: well-dispersed nanoparticles without inner channels/C (**A**); homogeneously dispersed porous nanoparticles/C (**B**); and porous nanoparticles/C dispersed nanoparticles forming a continuous phase/C hybrid membrane (**C**).

The enhancement of gas permeation in the hybrid CMS membranes is attributed to the reconstruction of the disordered ultramicropore structure in the pure carbon membrane by the incorporation of nanoparticles into the carbon matrix. Figure 23 illustrates the pore structure and gas diffusion routes model in the nanoparticles/C hybrid CMS membranes, in which the pore structure of hybrid CMS membrane is a composite of original ultramicropores, internal pores in porous doped nanoparticles, and interfacial pores created by the effect of micro-phase separation between the nanoparticle and polymeric precursors. The gas diffusion route includes the ultramicropores, interfacial pores and internal pore channels of porous nanoparticles. The interfacial pores are formed during the preparation of the mixed matrix membranes (MMMs) due to the effect of micro-phase separation [90]. The size of nanoparticles and chemical structure of the nanoparticle surface affect the size of interfacial pores and affinity between the nanoparticles and polymeric matrix [90,91,92]. The interfacial pores created around the nanoparticles were uniformly dispersed in the hybrid CMS membranes (shown in Figure 23A,B) and shrunk after carbonization, indicating that they only offer the fast diffusion routes of gas molecules to reduce the diffusion resistance through the CMS membrane and do not change the separation performance of CMS membranes.

As Figure 23A,B shows, the created new interfacial pores and internal pore channels of porous nanoparticles in a carbon matrix can provide a fast diffusion channel for gas molecules and reduce the gas diffusion resistance through the CMS membrane if the nanoparticles are properly loaded and well dispersed. The carbon matrix with an ultramicropore channel will act as a gas molecular sieve to maintain the high gas selectivity of the hybrid CMS membrane. However, the gas molecules will directly penetrate through the pore channel connected by the interfacial pores and internal pore of nanoparticles if the doping nanoparticles are overloaded and agglomerated in large sizes to form a continuous phase (Figure 23C), which will result in rapid reduction in gas selectivity of hybrid CMS membranes. That indicates that proper loading of nanoparticles is required for the fabrication of hybrid CMS membranes with higher gas permeability and selectivity. The microstructure and gas separation properties of hybrid CMS membranes can be controlled by tuning the membrane’s prepared conditions and the structure properties of hybridized nanoparticles.

## 6. Latest Developments in Hybrid CMS Membranes

In recent years, the gas permeability of pure CMS membranes has been greatly improved by use of high-performance CMS membrane precursors [93,94,95,96,97]. Research and attention on hybrid CMS membranes is less than before. However, it is undeniable that the preparation of hybrid CMS membranes is a very effective and simple method to prepare high-performance CMS membranes from the common, easily synthesized, low-cost precursors. If high performance precursor polymer matrix is doped with nano particles, the permeability of obtained CMS membranes may be further improved. If high selectivity can be maintained, the competitiveness of CMS membrane materials can be improved to a new level in the field of gas separation.

### 6.1. MOF/C Hybrid CMS Membrane

The metal-organic framework (MOF) materials can be carbonized at about 400 °C, and the formed nano-porous carbon is compatible with the carbon matrix in the CMS membrane. It implies that the porosity of CMS membranes could be reduced without being made denser, leading to an improved molecular sieving effect [98]. Jiao et al. [58] adopted ZIF-108 ((Zn(2-nitroimidazolate)2)), one of the MOF materials, with particle size of 160 nm as the novel dispersing nanoparticle and dispersed ZIF-108 into P84 polyimide as the polymer matrix to prepare the precursor of hybrid CMS membranes. After being pyrolyzed at 600 °C in Ar atmosphere, the ZIF-108/P84-based hybrid CMS membrane with higher gas permeability and selectivity (Table 12) than the pure P84-based CMS membrane was obtained. The doping ZIF-108 thermally degraded around 360 °C during the carbonization and formed nano carbons, which were compatible with the carbon matrix. As a result, the ultramicropore volume of the hybrid CMS membrane was much higher than the pure CMS membrane. The developed micropore structure could promote gas molecular permeation. The pore size distribution curve of the hybrid CMS membrane presented only one narrow ultramicropore peak with a diameter of 0.55 nm. Therefore, the molecular sieving effect was improved.

### 6.2. Fe/C hybrid CMS Membrane

Kumakiri et al. [99] used organosolve-lignin or phenol resin as the polymer matrix and mixed an iron(III) acetate base with the polymer to obtain the precursors. After dip-coating onto alumina tubes, drying and carbonizing, the composite Fe/C hybrid CMS membrane was produced. The organosolve-lignin or phenol resin-based hybrid CMS membrane with small amount of Fe compounds presented a similar H_2_ permeation rate to pure CMS membranes. However, other gas permeation rates were reduced and selectivity was improved. The Fe-based aggregations, which led crack formation, would be formed when the amount of Fe compounds was increased.

Fe particles in hybrid CMS membranes are too small to be observed. Hence, the interfacial gaps between particles and the carbon matrix which accelerated the gas permeation do not exist in these Fe/C hybrid CMS membranes. The pore structure of hybrid CMS membranes only depends on the effect of Fe on the carbon matrix during carbonization. The pore structure of hybrid CMS membranes with a small amount of Fe is more compact and similar to the pore structure in pure CMS membranes with a higher pyrolyzing temperature. For instance, both the pore size of the OrL-based CMS membrane carbonized at 700 °C (*P*_H2_ = 3.65 nmol m^−2^ s^−1^ Pa^−1^, *α*_H2/CH4_ = 630) and Fe/C hybrid CMS membranes carbonized at 500 °C (*P*_H2_ = 132 nmol m^−2^ s^−1^ Pa^−1^, *α*_H2/CH4_ = 584) are 0.41 nm, which is conductive to H_2_ permeation, according to the Knudsen-based permeance model. In our opinion, the primary catalytic graphitization led by Fe perhaps happened during the pyrolysis process, which promoted the formation of the carbon structure and made it more compact. Unfortunately, this is not demonstrated by Raman spectroscopy and other characterizations.

Separation of the gas pairs of C_2_H_4_/C_2_H_6_ and C_3_H_6_/C_3_H_8_ is an important process step in the petrochemical industry. Compared with the traditional distillation method with high energy-consumption, membrane separation method shows great advantages. The CMS membrane with molecular sieving capability has applied potential due to the very close dynamic diameters of these four gas molecules (C_2_H_4_ (3.75 Å) < C_3_H_6_ (3.82 Å) < C_2_H_6_ (3.85 Å) < C_3_H_8_ (3.95 Å)). Moreover, the different adsorption properties between the four gases could be used in separation. Chu et al. [100] were inspired by the special adsorption of olefins from the Fe^2+^ metal sites in Fe-MOF-74. They incorporated Fe(acac)_2_ of 1.1–3.2 wt% into the perfect CMS membrane precursor, 6FDA-DAM:DABA (3:2), which was independently researched and developed to prepare the Fe hybrid polymer membrane. After being carbonized at 550 °C or 675 °C, the Fe/C hybrid CMS membranes with good stability during long-term testing were obtained. The Fe^2+^ in Fe(acac)_2_ was cross-linked into the DABA-containing polymer and formed an average oxidation state of Fe^+2.1^ instead of oxidation to Fe^3+^ or reduction to Fe^0^. Therefore, the Fe^2+^ in the Fe/C hybrid CMS membranes acted the role of an active site to adsorb the C_2_H_4_. 

The highest selectivity, that is α_C2H4/C2H4_ = 8.53 ± 0.47 with P_C2H4_ ≈ 100 barrer, was presented in the Fe/C hybrid CMS membrane with Fe content of 2.2 wt% and carbonized at 550 °C. The pure CMS membrane with the same preparation condition showed P_C2H4_ > 1000 barrer and α_C2H4/C2H4_ ≈ 3. The sorption coefficients of C_2_H_4_ and C_2_H_6_ were tested, and the diffusion coefficients were calculated-based permeations. The Fe/C hybrid CMS membranes carbonized at 550 °C presented a 1.2 times higher sorption selectivity than the pure CMS membrane, implying the Fe complex in the micropores enhanced the sorption to olefins. Moreover, the diffusion selectivity of the hybrid CMS membrane is 2.7 times higher than pure CMS membranes. The reason is that the active Fe complex is attached to the edge of the carbon sheets and blocks some ultramicropores to increase the selective affinity to olefins. In this paper, a new, effective concept of incorporating Fe into the polymer to adjust the ultramicropore structure in CMS membranes and increase the diffusion selectivity of olefin or non-olefin/paraffin pairs was proposed. 

### 6.3. Boehmite/C Hybrid CMS Membrane

Llosa Tanco et al. [46,47] doped the boehmite nanoparticles into the novolac phenolic to synthesize the precursor. The boehmite was decomposed to Al_2_O_3_ above almost 400 °C. This phenomenon could make the pore structure more developed. The supported Al_2_O_3_/C hybrid CMS membrane with a CMS membrane thickness of ~3 μm was prepared by using the α-alumina tube as the support through a single dipping–drying–carbonization process (carbonization temperature from 450–1000 °C). The permeation rate first increased and then reduced due to the carbon structure forming and then shrinking with the carbonization temperature increasing. The highest permeations were observed for carbonization temperatures between 500 and 700 °C. However, these two papers did not mention any comparison of gas permeation property between the hybrid CMS membranes and pure CMS membrane without Al_2_O_3_ particles.

The vapor in the air greatly affected the gas permeation property of the Al_2_O_3_/C hybrid CMS membranes. Aging time (exposure time in ambient air) of 1 day would increase the selectivity of O_2_/N_2_ in the aged hybrid CMS membranes by 4.2 times and reduce the O_2_ permeation rate by 10.8 times compared with the fresh CMS membrane. This phenomenon became more significant over time because vapor reacted with reactive places of the membrane, implying oxygen chemisorption took place. Therefore, carbon containing the hydrophilic oxygen groups adsorb water by physical adsorption and by occupying some pores. The effective size of the micropores was reduced [46]. After regeneration by heat treatment at 100 to 200 °C under nitrogen, most of the initial performance of Al_2_O_3_/C hybrid CMS membrane was obtained.

The Al_2_O_3_/C hybrid CMS membrane carbonized at 550 °C with 1-day aging time showed H_2_/N_2_ selectivity of 725 and H_2_ permeance of 145 × 10^−9^ mol m^−2^ s^−1^ Pa^−1^ at room temperature, values comparable with the best performing Pd membranes [47]. This hybrid CMS membrane can therefore be used instead of the Pd hydrogen membrane for H_2_ separation processes at low temperatures [47].

## 7. The Evaluation of the Gas Separation Performance in the Hybrid CMS Membranes

**Figure 24:**
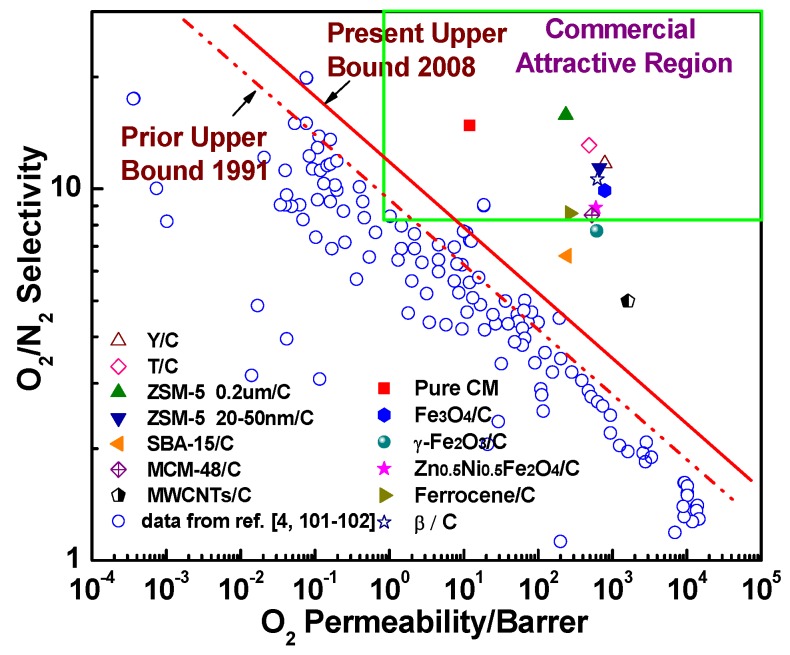
Permeability (O_2_) and selectivity (O_2_/N_2_) of hybrid CMS membranes.

**Figure 25:**
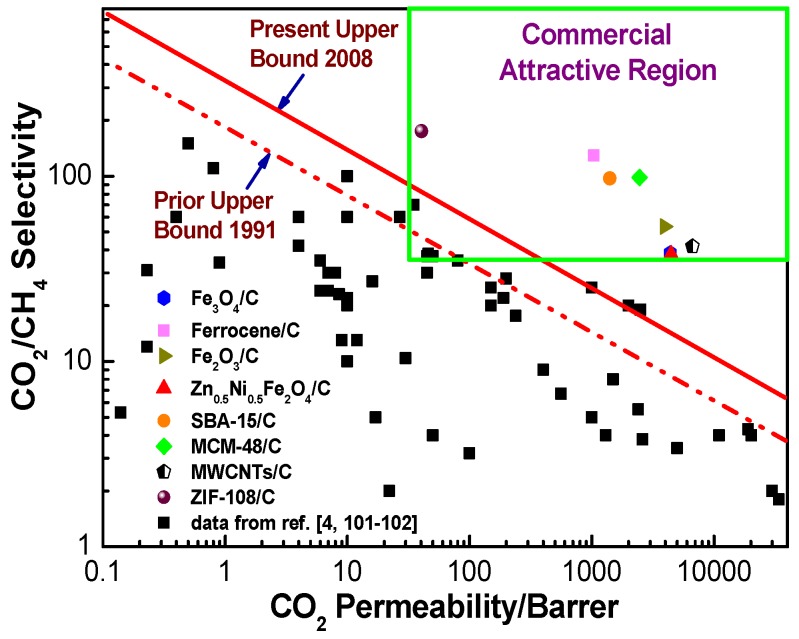
Permeability (CO_2_) and selectivity (CO_2_/CH_4_) of hybrid CMS membranes.

Robeson’s upper bounds proposed in 1991 [101] and 2008 [102] and commercially attractive regions suggested by Zhang et al. [26] and Hillock et al. [103], which are commonly recognized as convenient approaches to evaluate and exhibit the gas separation performance and commercial applicability of the membranes, were used to evaluate the gas separation performance of hybrid CMS membranes (Figure 24 and Figure 25). Compared to pure CMS membranes and polymeric membranes, the hybrid CMS membranes exhibit super high gas permeability with reasonable gas selectivity, which surpassed Robeson’s upper bounds and fell into the commercially attractive region. This suggested that incorporating nanoparticles into the carbon matrix can significantly enhance the gas permeability of derived hybrid CMS membranes with high gas selectivity if they are properly loaded. As Saufi and Ismail mentioned in their review [37], the high manufacture cost of pure CMS membranes, which is three orders of magnitude higher than polymer membranes, will see them lose their competitive ability over polymeric membranes even though pure carbon membranes possess outstanding gas selectivity. Therefore, the carbon membranes must achieve a superior gas separation performance, and extra high gas permeability, in particular, to compensate for their high cost and to increase their competitive ability over polymeric membranes in commercial applications. Hybrid CMS membranes, with their superior high gas permeability, have enhanced competitive ability, exhibiting their potential in commercial applications.

## 8. Conclusions and Prospects

The pure CMS membrane with chemical and thermal resistance presents outstanding selectivity in separating gas mixtures with very similar molecular size and higher permeability than polymeric membranes. However, the gas permeability of CMS membranes is still too low to satisfy the needs of commercial applications due to their higher cost compared to polymeric membranes. Enhancement of gas penetration while maintaining gas separation is the key to promoting the development and industrialization of CMS membranes. Optimizing the micropore structure of CMS membranes by doping inorganic nanoparticles into the carbon matrix to fabricate hybrid CMS membranes is an efficient and very simple method to improve the gas separation performance of CMS membranes. A series of inorganic particles, including Fe-related particles, zeolites, ordered porous silica and CNTs, are adopted to be incorporated into PAA and produce hybrid CMS membranes, which presented gas permeability that was two orders of magnitude higher than that of pure CMS membranes, while maintaining half the selectivity. The enhanced gas permeation of hybrid CMS membranes is attributed to the additional pore structure formed by the incorporation of particles into the carbon matrix, which includes the interfacial pores around the particles and inner pores of the particles. The carbon matrix maintains the molecular sieving effect for separation of the gases if the additional pores do not form a continuous phase in the hybrid CMS membranes. Compared to bigger particles with the same content, smaller particles tend to form smaller and more numerous interfacial pores. Hence, particles with smaller sizes or more developed inner pore structures are beneficial for preparing CMS membranes with higher gas permeability and selectivity. The gas separation property of the hybrid CMS membranes in this review have broken away from the “trade-off relationship” to be above Robeson’s upper bounds, and present commercially attractive potential in applications of air separation, CO_2_/CH_4_ separation, etc.

Some extensive further explorations based on this review are necessary:

- In recent years, some novel precursors, especially the novel polyimides with high fractional free volume and molecular chain rigidity [93,95,104] and PIMs [32,33], were synthesized to prepare CMS membranes with rather high gas permeability. It is necessary to improve the gas permeability further by doping the inorganic particles into these novel precursors to prepare the hybrid CMS membranes. More significantly, the preparation process should be investigated to prevent excessive reduction of selectivity.
- The hybrid CMS membranes related in this review are all self-standing flat membranes in order to study the intrinsic properties of the hybrid CMS membranes. However, coating the hybrid CMS membrane as a separation layer onto supports to fabricate supported CMS membranes is more valuable in practical applications. With a separation layer with a thickness of several micrometers, or even lower than 1 micrometer, the high gas permeability in free-standing hybrid carbon membranes would be transformed into the high gas permeation rate of supported CMS membranes. In particular, to ensure the high selectivity presented by the ultra-thin hybrid CMS membrane separation layer, not only should the proper coating methods be adopted to prevent cracks and pinholes, more importantly, doping particles with as small a size as possible and homogenous dispersion are necessary to prevent the formation of connected gas penetrating channels through the separation layer.
- The hybrid CMS membranes in this review with more outstanding gas permeation properties can be used in all the applied fields of pure CMS membranes. Recently, in addition to conventional gas separation, pure CMS membranes have presented excellent performance in the field of membrane reactors and attracted much attention of researchers. Itoh et al. [10] applied CMS membranes to the dehydrogenation of cyclohexane to remove the produced H_2_ and improved the conversion rate from 30% to 70%. Zhang et al. [11] used the CMS membrane reactor to the methanol steam reforming reaction to produce H_2_ with the methanol conversion rate as high as 99.9% and H_2_ selectivity of 97% at 250 °C. Briceño et al. [105,106] and Zhang et al. [107] also researched the methanol steam reforming reaction with CMS membrane reactor and achieved higher conversions with the CMS membrane reactor than the traditional reactors. Abdollahi et al. [12] took coal-based syngas containing H_2_S as a raw material and achieved a synchronous separation reaction to obtain H_2_ in the CMS membrane reactor. Hirota et al. [9] prepared gas-activated CMS membranes with outstanding H_2_ permeation and selectivity of H_2_/meth cyclohexane, which presented the potential in H_2_ storage. Hybrid CMS membranes have better application prospects than pure CMS membranes in the field of membrane reactors because the doping nanoparticles have catalytic activity and can replace some additional catalysts, in addition to the benefit of the excellent gas permeation performance of the hybrid CMS membranes.

## Figures and Tables

**Table 1:** Gas permeability and selectivity for some nanoparticle/C hybrid membranes.

Sample	Permeability/Barrer ^a^					Ideal Selectivity			
	H_2_	CO_2_	O_2_	N_2_	CH_4_	O_2_/N_2_	CO_2_/N_2_	CO_2_/CH_4_	H_2_/N_2_
Ag/C [42]	-	290	81.3	6.7	-	12.1	43.3	--	-
K/C [60]	-	-	6.8	1.7	-	4	-	-	-
Na/C [60]	-	-	5.3	1.1	-	4.8	-	-	-
Pd based [40]	34.4	-	-	0.0061	-	-	-	-	5639.3
CaO/C [45]	860	130	38	3.1	3.5	12.3	41.9	37.1	277.4
FeO [45]	280	110	30	8.3	4	3.6	13.3	27.5	33.7
AgN/C [45]	1500	180	53	5.1	1.4	10.4	352.9	1285.7	294.1

^a^ 1 barrer = 1 × 10^−10^ cm^−3^(STP) cm/cm^2^ s cmHg (STP: standard temperature and pressure).

**Table 2:** Gas permeability and selectivity for ferrocene/PAA-based Fe/C hybrid membranes [63] (Testing condition: 0.05 MPa, 25 °C, the same below).

Sample	Permeability/Barrer					Ideal Selectivity			
	H_2_	CO_2_	O_2_	N_2_	CH_4_	O_2_/N_2_	CO_2_/N_2_	CO_2_/CH_4_	H_2_/N_2_
CMSM ^a^ [59]	84.4	52.7	4	0.27	-	14.8	195	-	312.6
Ferrocene 10%	1789	634	159	16	3	9.9	39.6	211.0	111.8
Ferrocene 15%	2806	1039	266	31	8	8.6	33.5	129.5	90.5
Ferrocene 20%	6997	2275	1264	1001	1413	1.3	1.8	1.6	7.0

^a^ CMSM = Pure CMS membrane prepared in the same condition.

**Table 3:** Gas permeability and ideal selectivity of Fe series magnetic nanoparticle/C hybrid membranes.

Sample	Permeability/Barrer					Ideal Selectivity			
	H_2_	CO_2_	O_2_	N_2_	CH_4_	O_2_/N_2_	CO_2_/N_2_	CO_2_/CH_4_	H_2_/N_2_
CMSM [59]	84.4	52.7	4	0.27	-	14.8	195	-	312.6
Fe_3_O_4_ 10% [64]	6790	2764	786	79	48	9.9	35.0	57.6	85.9
Fe_3_O_4_ 15% [64]	12,194	3433	1175	136	74	8.6	25.2	46.4	89.7
Fe_3_O_4_ 20% [64]	15,476	4385	1565	193	114	8.1	22.7	38.5	80.2
γ-Fe_2_O_3_ 10%	5415	2376	616	80	36	7.7	29.7	66.0	67.7
γ-Fe_2_O_3_ 15%	7752	2790	643	86	48	7.5	32.4	58.1	90.1
γ-Fe_2_O_3_ 20%	8035	3954	1187	166	74	7.2	23.8	53.4	48.4
Zn_0.5_Ni_0.5_Fe_2_O_4_ 10%	5814	2401	599	67	27	8.9	35.8	88.9	86.8
Zn_0.5_Ni_0.5_Fe_2_O_4_ 15%	6162	2784	690	85	39	8.1	32.8	71.4	72.5
Zn_0.5_Ni_0.5_Fe_2_O_4_ 20%	8191	4466	1180	181	118	6.5	24.7	37.8	45.3
Pd based [40]	34.4	-	-	0.0061	-	-	-	-	5639.3
CMSM in ref. [40]	49.4	-	-	0.15	-	-	-	-	329.3
Ag/PFR based [42]	-	290	81.3	6.7	-	12.1	43.3	-	-
CMSM in ref. [42]	-	64.1	16.8	2.1	-	8	30.5	-	-

**Table 4:** The effect of zeolite content in the precursor on the gas separation performance of the zeolite/C hybrid membranes [59].

Sample	Content (wt%)	Permeability (Barrer)					Ideal Selectivity			
		H_2_	CO_2_	O_2_	N_2_	CH_4_	H_2_/N_2_	CO_2_/N_2_	O_2_/N_2_	CO_2_/CH_4_
CMSM		84.4	52.7	4	0.27	-	312.6	195.1	14.8	-
β/C	5	543	255	66	5.6	-	97	45.5	11.8	-
	10	1721	810	274	21.3	-	80.8	38	12.9	-
	15	2108	1129	382	27.9	-	75.6	40.5	13.7	-
	20	2987	1360	493	42.5	-	70.3	32	11.6	-
	25	3996	1644	628	59.4	-	67.3	27.7	10.6	-
Y/C	5	561	250	55	5.6	-	100.2	44.6	9.8	-
	10	1717	761	236	20.7	-	82.9	36.8	11.4	-
	15	2280	1022	501	32.1	-	71	31.8	15.6	-
	20	3090	1431	605	45.5	-	67.9	31.5	13.3	-
	25	4094	1783	786	67.1	-	61	26.6	11.7	-
Y/C [52]		-	266	-	-	2.15	-	-	-	124
CMSM [52]		-	611	-	-	10	-	-	-	61

**Table 5:** The effect of crystal diameter on the gas separation performance of the zeolite/C hybrid membranes (zeolite content in precursor of 10 wt% unless otherwise indicated).

Sample	Diameter of Single Crystal	Permeability (Barrer)				Ideal Selectivity		
		H_2_	CO_2_	O_2_	N_2_	H_2_/N_2_	CO_2_/N_2_	O_2_/N_2_
T/C [57]	0.5 μm	4230	1773	486	37.2	113.7	47.7	13.1
	3 μm	2474	1580	341	41.5	59.6	38.1	8.2
	6 μm	4094	1578	349	46.5	88	33.9	7.5
	8 μm	4168	2151	492	107	39	20.1	4.6
ZSM-5/C [59]	100 nm	1179	564	237	15.0	78.6	37.7	15.8
	1 μm	1366	629	253	23.6	57.9	26.6	10.7
	5 μm	1447	637	254	27.0	53.6	23.7	9.4
	10 μm	1506	660	257	30.1	50	22	8.5

**Table 6:** The effect of agglomeration degree on the gas separation performance of the ZSM-5/C hybrid membranes.

Agglomeration Particle Diameter	Permeability (Barrer)				Ideal Selectivity		
	H_2_	CO_2_	O_2_	N_2_	H_2_/ N_2_	CO_2_/N_2_	O_2_/N_2_
20–50 nm 9.09 wt% [56]	5399	3020	671	59	91.5	51.2	11.4
0.2 μm [59]	1179	564	237	15.0	78.6	37.7	15.8
1 μm [59]	1190	607	221	18.2	65.4	33.4	12.1
4 μm [59]	1224	624	231	21.6	56.7	28.9	10.7

**Table 7:** The effect of carbonization temperature on the gas separation performance of the zeolite/C hybrid membranes and pure CMS membrane [59].

Membrane	Carbonization Temperature (°C)	Permeability (Barrer)				Selectivity		
		H_2_	CO_2_	O_2_	N_2_	H_2_/N_2_	CO_2_/N_2_	O_2_/N_2_
CMSM	600	16.5	6.57	0.74	0.05	330	123	13.8
	700	84.4	52.7	4	0.27	312.6	195	14.8
	800	36	25	1.71	0.11	327.3	234	16
zeolite β/C hybrid membrane	600	2234	1303	357	51.9	43	25.1	6.9
	700	1721	810	274	21.3	80.8	38	12.8
	800	253	158	12	0.81	312.3	195	14.8
zeolite Y/C hybrid membrane	600	2304	1148	298	50.8	45.4	22.6	5.86
	700	1717	761	236	20.7	82.9	36.7	11.4
	800	102	42.6	7.5	0.7	145.7	59.3	10.5

**Table 8:** The effect of channel integrity on the gas separation performance of the zeolite Y/carbon hybrid membranes [59].

Sample	Permeability (Barrer)				Ideal Selectivity		
	H_2_	CO_2_	O_2_	N_2_	H_2_/N_2_	CO_2_/N_2_	O_2_/N_2_
Y (pyrolyzed)/carbon	339	204	38	4	84.8	51	9.6
Y (intact)/carbon	1717	761	236	20.7	82.9	36.7	11.4
CMSM	84.4	52.7	4	0.27	312.6	195.1	14.8

**Table 9:** Gas permeability and selectivity of pure CMS membrane and ordered mesoporous silica/C hybrid CMS membranes.

Sample	Permeability/Barrer					Ideal Selectivity		
	H_2_	CO_2_	O_2_	N_2_	CH_4_	O_2_/N_2_	CO_2_/N_2_	CO_2_/CH_4_
CMSM [59]	84.4	52.7	4	0.27	-	14.8	195.1	-
500 nm SBA-15/C [38]	1807	1410	246	37	14.5	6.6	38.1	97.2
100–200 nm MCM-48/C [38]	3838	2461	527	62	25	8.5	39.7	98.4

**Table 10:** Gas permeability and selectivity of pure CMS membrane and C/CNT hybrid membranes [89].

Sample		Permeability/Barrer					Ideal Selectivity			
		H_2_	CO_2_	O_2_	N_2_	CH_4_	O_2_/N_2_	CO_2_/N_2_	H_2_/N_2_	CO_2_/CH_4_
CMSM		853	321	151	14	6	10.79	22.93	60.93	53.50
SWCNT/C		2563	2626	522	96	46	5.4	27.4	26.7	57.1
MWCNT2/C		4931	6659	1307	280	206	4.7	23.8	17.6	32.3
MWCNT2 ^1^ (Acid treated)/C		5824	6661	1259	253	161	5.0	26.3	23.0	41.4
MWCNT2 Concentration	0	853	321	151	14	6	10.8	22.9	60.9	53.5
	5%	2600	3407	701	139	107	5.1	24.3	18.7	31.8
	10%	5824	6661	1259	253	161	5.0	26.3	23.0	41.4
	15%	7071	9332	1576	335	256	4.7	27.9	21.1	36.5
MWCNT1 ^2^/C		4659	5425	1061	234	204	4.5	23.2	19.9	26.6
MWCNT3 ^3^/C		3886	4395	851	164	103	5.2	26.8	23.7	42.7

^1^ MWCNT2: diameter = 40–60 nm, length = 1–2 μm. ^2^ MWCNT1: diameter = 40–60 nm, length = 5–15 μm. ^3^ MWCNT3: diameter = 10–20 nm, length = 5–15 μm.

**Table 11:** Gas permeability and selectivity of pure CMS membrane and representative hybrid CMS membranes.

Sample or Hybridized Particle (Content in the Precursor)	Permeability/Barrer ^a^					Ideal Selectivity			
	H_2_	CO_2_	O_2_	N_2_	CH_4_	O_2_/N_2_	CO_2_/N_2_	H_2_/N_2_	CO_2_/CH_4_
CMSM [59]	84.4	52.7	4	0.27	-	14.8	195.1	312.6	-
MWCNT2 (10%) [89]	5824	6661	1259	253	161	5.0	26.3	23.0	41.4
500 nm SBA-15 (10%) [38]	1807	1410	246	37	14.5	6.6	38.1	48.8	97.2
100–200 nm MCM-48 (10%) [38]	3838	2461	527	62	25	8.5	39.7	61.9	98.4
0.5 μm T (10%) [57]	4230	1773	486	37.2	-	13.1	47.7	113.7	-
100 nm ZSM-5 (10%) [59]	1179	564	237	15.0	-	15.8	37.6	78.6	-
20–50 nm ZSM-5 (9.09 wt. %) [56]	5399	3020	671	59	-	11.4	51.2	91.5	-
20–50 nm Fe_3_O_4_ (10%) [64]	6790	2764	786	79	48	9.9	35.0	86.0	57.6

**Table 12:** Mixed gas permeability and separation factor for P84-based CMS membrane and hybrid CMS membrane with ZIF-108/P84 = 0.1 (testing condition: 0.1 MPa and 25 °C) [58].

Sample	CO_2_ Permeability/Barrer	CO_2_/CH_4_ Separation Factor
P84-based CMS membrane	7.1	72.4
Hybrid CMS membrane with ZIF-108/P84 = 0.1	40.9	174
